# The Role of Herbal Medicines in Cognitive Impairment: A Narrative Review of Neuroprotective Mechanisms and Therapeutic Perspectives

**DOI:** 10.3390/nu18193126

**Published:** 2026-09-23

**Authors:** Aekkhaluck Intharuksa, Anchalee Prasansuklab, Tachpon Techarang, Kamonwan Chaikhong, Warunya Arunotayanun, Rewadee Jenraumjit

**Affiliations:** 1 Department of Pharmaceutical Sciences, Faculty of Pharmacy, Chiang Mai University, Chiang Mai 50200, Thailand; aekkhaluck.int@cmu.ac.th; 2 College of Public Health Sciences, Chulalongkorn University, Bangkok 10330, Thailand; anchalee.pr@chula.ac.th; 3 Center of Excellence on Natural Products for Neuroprotection and Anti-Ageing, Chulalongkorn University, Bangkok 10330, Thailand; kamonwan.chaikhong@gmail.com; 4 Department of Tropical Pathology, Faculty of Tropical Medicine, Mahidol University, Bangkok 10400, Thailand; tachpon.tec@mahidol.ac.th; 5 Kanchanabhishek Institute of Medical and Public Health Technology, Faculty of Public Health and Allied Health Science, Praboromarajchanok Institute, Nonthaburi 11150, Thailand; warunya@kmpht.ac.th; 6 Department of Pharmaceutical Care, Faculty of Pharmacy, Chiang Mai University, Chiang Mai 50200, Thailand

**Keywords:** cognition, memory impairment, mild cognitive impairment, dementia, Alzheimer’s disease, herbal medicines, neuroprotection

## Abstract

Cognitive impairment encompasses a clinical spectrum ranging from mild cognitive impairment to dementia, particularly Alzheimer’s disease (AD), and involves interconnected pathological processes including cholinergic dysfunction, oxidative stress, neuroinflammation, mitochondrial impairment, amyloid-β accumulation, tau hyperphosphorylation, and synaptic loss. Herbal medicines have attracted increasing attention because their complex phytochemical composition may provide multi-target neuroprotective effects. This narrative review aimed to summarize current preclinical and clinical evidence on herbal medicines and traditional herbal formulations for cognitive impairment, with emphasis on their mechanisms, therapeutic potential, safety, and translational limitations. Relevant evidence from in vitro studies, animal models, randomized clinical trials, systematic reviews, and meta-analyses was narratively synthesized. The review considered single herbal medicines, standardized extracts, isolated phytochemicals, and traditional multi-herbal formulations, together with their reported cognitive outcomes and molecular targets. Preclinical studies suggest that selected herbal preparations may modulate pathways implicated in cognitive impairment through acetylcholinesterase and butyrylcholinesterase inhibition, antioxidant and mitochondrial protection, suppression of neuroinflammatory signaling, modulation of amyloidogenic and tau-related pathways, and enhancement of BDNF/TrkB- and CREB-associated neuroplasticit; however, clinical evidence remains limited and heterogeneous. Clinical studies of *Bacopa monnieri*, *Panax ginseng*, *Crocus sativus*, and several traditional Chinese, Korean, and Persian formulations have reported improvements in memory and global cognitive measures, including MMSE, MoCA, and ADAS-cog scores. However, evidence remains constrained by heterogeneity of products, small sample sizes, short treatment periods, inconsistent outcome measures, limited biomarker integration, and incomplete long-term safety assessment. Herbal medicines show potential as preventive, complementary, or adjunctive approaches for cognitive impairment, but they cannot yet be considered established replacements for conventional therapy. Standardized products, rigorous pharmacokinetic and toxicological evaluation, disease-relevant models, and adequately powered multicenter trials are required to support evidence-based clinical application.

## 1. Introduction

Cognitive decline, ranging from Mild Cognitive Impairment (MCI) to severe Dementia, has emerged as one of the most significant global health challenges of the 21st century. As the global population ages, the prevalence of neurodegenerative disorders, particularly Alzheimer’s Disease, is projected to increase exponentially. It is estimated that over 55 million people worldwide are currently living with dementia, a figure expected to rise to 139 million by 2050 [1]. These conditions not only diminish the quality of life for patients but also impose a profound socioeconomic burden on healthcare systems and caregivers globally.

### 1.1. Definition

According to the American Psychiatric Association (2013), dementia, or Major Neurocognitive Disorder (major NCD), is not a specific disease but an umbrella term for a clinical syndrome characterized by a significant decline in one or more cognitive domains (e.g., memory, planning, calculation, executive function, language, decision-making, or social cognition) [2]. In this context, the diagnosis of dementia requires not only measurable cognitive deficits but also evidence of functional impairment in daily life. Patients with dementia typically experience impairment in at least two functions, with a greater decline than their peers. This decline must represent progressively from previous performance and, crucially, must be severe enough to interfere with independence in everyday activities (e.g., managing finances or medications) [2,3].

Based on these diagnostic characteristics, dementia can be classified into several major subtypes according to its underlying etiology and clinical features. There are several types of dementia including are Alzheimer’s disease (AD), vascular dementia (VD), Lewy body dementia (LBD), and Frontotemporal lobe dementia (FTD). Among these, AD is the most prevalent and has been extensively studied due to its substantial global burden. This review will focus on AD, the most common type, accounting for approximately 60% of cases [4]. AD is considered a major NCD characterized by impairment in at least two cognitive domains, memory and at least one other cognitive domain must be affected. This impairment must progressively worsen to the point of significantly impacting mood, behavior, caregiver burden and overall quality of life [5].

Importantly, cognitive decline exists along a continuum, and not all individuals with cognitive impairment meet the criteria for dementia. Rather than a simple obligate transitional phase, mild cognitive impairment (MCI) represents a clinically heterogeneous syndrome characterized by objective cognitive impairment in one or more domains with relatively preserved functional independence in daily activities [6]. Although individuals with MCI exhibit an increased risk of progressing to dementia, this progression is not inevitable; substantial subsets of patients remain clinically stable for extended periods, and some may even revert to normal cognition.

### 1.2. Pathology and Pathophysiology of Cognitive Impairment

Cognitive impairment, particularly in AD, is characterized by the accumulation of amyloid-β plaques and hyperphosphorylated tau neurofibrillary tangles, accompanied by synaptic loss, neuronal degeneration, and progressive brain atrophy in key regions such as the hippocampus and cerebral cortex [6]. These pathological changes begin years before clinical onset, reflecting a prolonged preclinical phase. The disease is multifactorial, involving protein aggregation, neuroinflammation, oxidative stress, mitochondrial dysfunction, excitotoxicity, and vascular impairment, which collectively disrupt synaptic function and neuroplasticity, leading to neuronal loss and cognitive decline [7,8]. The major genetic and molecular influences, pathological hallmarks, and interconnected pathophysiological mechanisms contributing to cognitive impairment in AD are summarized in Figure 1. This complexity has driven increasing interest in multi-target therapeutic strategies.

#### 1.2.1. Classification and Clinical Spectrum of Cognitive Impairment

Cognitive impairment exists along a continuum from normal aging to MCI and ultimately to dementia. This progression reflects the gradual accumulation of neuropathological changes and failure of compensatory neural mechanisms. MCI is characterized by measurable cognitive decline without significant impairment in daily functioning and represents a high-risk state for progression to dementia, particularly AD [9]. Dementia is defined by progressive deterioration in multiple cognitive domains that interfere with independence. AD accounts for the majority of cases, although mixed pathologies are frequently observed [10]. Whereas non-Alzheimer dementias often present initially with motor, behavioral, or executive dysfunction, Alzheimer’s disease is predominantly defined by insidious episodic memory loss driven by hallmark amyloid and tau pathology. Recent biomarker-based frameworks emphasize the biological definition of AD, incorporating amyloid, tau, and neurodegeneration markers [11]. This paradigm shift highlights that cognitive impairment is not merely a clinical syndrome but reflects underlying molecular pathology. Furthermore, when conceptualizing cognitive impairment, a clear distinction must be maintained between the overarching clinical syndrome of dementia—defined by functional dependence across multiple cognitive domains—and the etiological or biological diagnosis of Alzheimer’s disease (AD). While AD is the leading underlying cause of dementia, contemporary diagnostic frameworks define AD biologically by the presence of specific neuropathological hallmarks (e.g., amyloid-β and hyperphosphorylated tau biomarkers), which can exist long before the onset of overt clinical dementia or even in individuals presenting primarily with MCI.

#### 1.2.2. Genetic and Molecular Influences

1. APOE ε4

The apolipoprotein E (APOE) ε4 allele represents the most significant genetic risk factor for late-onset AD and plays a central role in modulating multiple pathogenic pathways has been proposed to modulate associated with cognitive impairment [12,13]. APOE is a lipid transport protein primarily produced by astrocytes in the central nervous system and is essential for cholesterol homeostasis, synaptic repair, and neuronal maintenance [13]. Among the three major isoforms (ε2, ε3, and ε4), the ε4 variant is robustly associated susceptibility to AD in a gene dose-dependent manner [12]. Individuals carrying one ε4 allele exhibit a 3- to 4-fold increased risk, whereas homozygous carriers (ε4/ε4) may have up to a 12-fold increased risk and earlier disease onset [12]. Mechanistically, APOE ε4 may contribute to cognitive impairment through several interconnected processes. In experimental models, it has been shown to facilitate amyloid-β (Aβ) aggregation and reduces its clearance across the blood–brain barrier, potentially by impairing receptor-mediated transport pathways such as low-density lipoprotein receptor-related protein 1 (LRP1) [13,14]. These actions may promote extracellular Aβ deposition and plaque formation [13]. In addition, APOE ε4 has been associated with altered lipid metabolism, resulting in reduced synaptic repair and impaired membrane remodeling, which are critical for neuronal plasticity [14]. Furthermore, preclinical evidence suggests that *APOE* ε4 may influence tau-mediated neurodegeneration independently of amyloid pathology and enhances neuroinflammatory responses [14]. It has also been implicated in mitochondrial dysfunction and increased oxidative stress, processes that have been proposed to exacerbate neuronal vulnerability and accelerated cognitive decline [13,14].

2. TREM2 and Microglial Function

Triggering receptor expressed on myeloid cells 2 (TREM2) is a transmembrane receptor predominantly expressed on microglia and plays a crucial role in regulating innate immune responses in the brain [15]. TREM2 participates in microglial activation, phagocytosis, lipid sensing, and survival, thereby modulating central nervous system homeostasis [15]. Genetic variants in TREM2, particularly the R47H mutation, have been proposed to identify as important risk factors for AD, conferring a 2- to 3-fold increased risk [16]. These variants are associated with altered microglial function and a compromised capacity to process amyloid-β and cellular debris [15]. At the mechanistic level, TREM2 signaling is required for the transition of microglia toward a mature disease-associated microglia (DAM) phenotype and promotes microglial clustering around amyloid-β plaques. In experimental models, this plaque-associated microglial response has been shown to increase plaque compaction and limit local neuritic damage, particularly during early stages of amyloid deposition [15,17,18]. However, recent evidence indicates that microglial phenotypes and TREM2-mediated signaling are highly heterogeneous and context-dependent rather than uniformly beneficial. While intact TREM2 signaling promotes microglial clustering and amyloid plaque compaction, its effects may differ at later disease stages or in the presence of established tau pathology and chronic neuroinflammation. In experimental tauopathy models, reduced TREM2 signaling has been associated with attenuated neuroinflammation and neurodegeneration, highlighting a stage- and pathology-dependent role for this pathway [19]. More broadly, persistently activated microglia may contribute to maladaptive synaptic engulfment and sustained pro-inflammatory signaling [20]. Conversely, TREM2 loss-of-function or haplodeficiency impairs the plaque-associated microglial barrier, leading to reduced amyloid compaction, more diffuse plaque morphology, and increased neuritic or axonal dystrophy in amyloid models [18,21]. In addition to impaired phagocytosis, TREM2 deficiency leads to dysregulated inflammatory signaling. Microglia lacking functional TREM2 exhibit altered cytokine production and reduced survival, contributing to a chronic inflammatory environment that can exacerbate synaptic dysfunction and neuronal loss [15]. TREM2 has been associated with lipid metabolism and energy regulation within microglia, linking metabolic dysfunction to neurodegenerative processes [15].

3. Neurotrophic Factors (BDNF)

Brain-derived neurotrophic factor (BDNF) is a key regulator of neuronal survival, synaptic plasticity, and memory formation [22,23]. It is highly expressed in the hippocampus and cortex and plays a critical role in long-term potentiation (LTP), which underlies learning and memory processes [23]. Reduced BDNF expression has been consistently observed in individuals with cognitive impairment, mild cognitive impairment, and AD [22]. This reduction contributes to impaired synaptic plasticity, decreased neurogenesis, and increased neuronal vulnerability, ultimately leading to memory impairment and cognitive decline [22,23]. At the molecular level, BDNF exerts its effects through activation of the tropomyosin receptor kinase B (TrkB), triggering downstream signaling pathways such as PI3K/Akt, MAPK/ERK, and PLCγ [23]. Dysregulation of these pathways results in impaired neuronal survival and synaptic dysfunction [23]. Genetic variation in the BDNF gene, particularly the Val66Met polymorphism, affects activity-dependent secretion of BDNF [24]. Individuals carrying the Met allele exhibit reduced hippocampal volume, impaired memory performance, and increased susceptibility to neurodegeneration [24]. Additionally, BDNF expression is influenced by environmental factors such as physical activity, stress, and diet, suggesting a role in modulating cognitive resilience [23].

#### 1.2.3. Pathological Hallmarks of Cognitive Impairment

1. Amyloid-β Deposition

Amyloid-β (Aβ) accumulation is a major pathological hallmark of AD and has traditionally been considered a key initiating role in the amyloid cascade hypothesis [25]. Aβ peptides are generated from amyloid precursor protein (APP) through sequential cleavage by β-secretase (BACE1) and γ-secretase, producing peptides of varying lengths, among which Aβ1–42 is particularly prone to misfolding and aggregation due to its hydrophobic nature [25,26]. These peptides initially assemble into soluble oligomers, which can further aggregate into protofibrils and eventually form insoluble extracellular plaques [26]. Increasing evidence indicates that soluble Aβ oligomers, rather than mature fibrillar plaques, represent a primary driver of early synaptic dysfunction [26,27]. These oligomers can interfere with synaptic signaling by disrupting glutamatergic neurotransmission, altering NMDA receptor activity, and impairing long-term potentiation (LTP), a fundamental mechanism underlying learning and memory [27]. At the cellular level, Aβ oligomers contribute to calcium dysregulation, mitochondrial dysfunction, and oxidative stress, ultimately promoting synaptic loss and neuronal injury [27]. They also interact with multiple receptors, including cellular prion protein (PrPC) and NMDA receptors, further amplifying neurotoxicity [27]. Importantly, Aβ accumulation begins years to decades before clinical symptoms appear, highlighting its role in the preclinical phase of cognitive impairment, although it represents one critical component within a broader, multifactorial cascade involving tau pathology, neuroinflammation, and neurodegeneration.

2. Tau Pathology and Neurofibrillary Tangles

Tau is a microtubule-associated protein that stabilizes microtubules and maintains axonal transport in neurons [28]. Under pathological conditions, tau undergoes abnormal hyperphosphorylation mediated by kinases such as glycogen synthase kinase-3β (GSK-3β) and cyclin-dependent kinase 5 (CDK5), resulting in reduced binding affinity to microtubules [28,29]. This detachment leads to microtubule destabilization and impaired axonal transport, ultimately contributing to neuronal dysfunction [28]. Hyperphosphorylated tau aggregates into paired helical filaments that form intracellular neurofibrillary tangles (NFTs), one of the defining pathological features of AD [29]. The progression of tau pathology follows a highly ordered spatial pattern described by Braak staging, beginning in the transentorhinal cortex, spreading to the hippocampus, and eventually involving widespread neocortical regions [30]. Notably, the distribution and burden of tau pathology correlate more strongly with the severity of cognitive impairment than amyloid deposition, suggesting a more direct role in neuronal dysfunction and clinical symptoms [29,30]. In addition to structural disruption, pathological tau can propagate between neurons in a prion-like manner, facilitating the spread of neurodegeneration across brain networks [29]. This propagation contributes to progressive synaptic failure and cognitive decline.

3. Synaptic Loss and Cortical Atrophy

Synaptic dysfunction and loss represent the strongest pathological correlates of cognitive impairment and are more closely associated with cognitive decline than either amyloid plaques or neurofibrillary tangles alone [31]. Early synaptic alterations include reduced dendritic spine density, impaired synaptic transmission, and disruption of synaptic plasticity mechanisms such as long-term potentiation (LTP) [6,31,32]. These changes are driven by the combined effects of amyloid-β toxicity, tau pathology, neuroinflammation, and oxidative stress, which collectively impair neuronal communication and network integrity [31]. Aβ oligomers directly target synapses, while tau pathology disrupts axonal transport and synaptic stability, leading to progressive synaptic degeneration [32]. At the macroscopic level, these processes manifest as cortical atrophy, particularly in regions critical for cognition, including the hippocampus, entorhinal cortex, and association cortices [33]. Neuroimaging studies in individuals with mild cognitive impairment and dementia consistently demonstrate volume loss in these regions, correlating with deficits in memory, attention, and executive function [33]. Ultimately, synaptic loss leads to disconnection of neural networks, impairing information processing and integration across brain regions. This network failure underlies the clinical manifestations of cognitive impairment and represents a key endpoint of multiple converging pathological processes.

#### 1.2.4. Pathophysiological Mechanisms

1. Oxidative Stress and Mitochondrial Dysfunction

Oxidative stress arises from an imbalance between reactive oxygen species (ROS) production and antioxidant defense systems and is a key contributor to neuronal damage in cognitive impairment [8]. Neurons are particularly susceptible to oxidative stress due to their high oxygen consumption, abundant lipid content, and relatively low antioxidant capacity [8]. Mitochondrial dysfunction plays a central role in this process by impairing ATP production and increasing ROS generation through defects in the electron transport chain [34]. Accumulation of amyloid-β within mitochondria further disrupts mitochondrial function, leading to impaired energy metabolism and increased oxidative burden [34]. This creates a vicious cycle in which ROS-induced mitochondrial damage further enhances ROS production. Oxidative modifications of proteins, lipids, and nucleic acids, such as lipid peroxidation, protein carbonylation, and DNA oxidation, impair neuronal function and synaptic signaling [8]. These alterations contribute to synaptic failure, activation of apoptotic pathways, and progressive neurodegeneration, ultimately leading to cognitive decline.

2. Excitotoxicity

Glutamate-mediated excitotoxicity is a critical mechanism underlying neuronal injury and loss in cognitive impairment [35]. Under pathological conditions, excessive extracellular glutamate accumulates due to impaired reuptake by astrocytes and dysregulated synaptic release [35]. Overactivation of N-methyl-D-aspartate (NMDA) receptors leads to excessive calcium influx into neurons, triggering a cascade of deleterious events including mitochondrial dysfunction, activation of proteases and endonucleases, and generation of reactive oxygen species [34,35]. This ultimately results in neuronal apoptosis and synaptic loss. Excitotoxic damage is particularly prominent in hippocampal neurons, which are highly sensitive to calcium dysregulation and play a central role in memory formation [35]. Pharmacological modulation of NMDA receptors using non-competitive antagonists such as memantine provides partial neuroprotection by reducing pathological receptor activation while preserving physiological signaling [35].

3. Neuroinflammation

Neuroinflammation is a central component of cognitive impairment and plays both protective and detrimental roles depending on disease stage [7]. In early stages, microglial activation may facilitate the clearance of amyloid-β deposits through phagocytosis [36]. However, chronic activation of microglia and astrocytes leads to sustained release of pro-inflammatory mediators, including tumor necrosis factor-α (TNF-α), interleukin-1β (IL-1β), and interleukin-6 (IL-6), contributing to a neurotoxic environment [7]. This persistent inflammatory state promotes synaptic dysfunction, dendritic spine loss, and neuronal injury, ultimately impairing cognitive function [7,36]. Moreover, neuroinflammation accelerates tau pathology by enhancing tau phosphorylation and aggregation through kinase activation (e.g., GSK-3β and CDK5) [36]. Dysregulated microglial responses, influenced by genetic factors such as TREM2, further exacerbate disease progression by impairing amyloid clearance and promoting chronic inflammation [16,36]. Collectively, these processes establish a feed-forward loop between inflammation and neurodegeneration.

4. Cholinergic Deficits

The cholinergic hypothesis posits that degeneration of basal forebrain cholinergic neurons, particularly in the nucleus basalis of Meynert, leads to reduced acetylcholine (ACh) levels in the cortex and hippocampus regions critically involved in cognition [37,38]. Acetylcholine plays a fundamental role in modulating attention, learning, synaptic plasticity, and memory encoding through both muscarinic and nicotinic receptor signaling [38]. Loss of cholinergic signaling results in impaired long-term potentiation (LTP), reduced cortical excitability, and disrupted hippocampal-dependent memory processes [37]. Additionally, cholinergic deficits are associated with increased amyloid-β deposition and enhanced tau phosphorylation, suggesting a bidirectional relationship between neurotransmitter dysfunction and core Alzheimer’s pathology [39]. This mechanistic link forms the basis for current symptomatic treatments using acetylcholinesterase inhibitors such as donepezil, rivastigmine, and galantamine, which transiently improve cognitive function by increasing synaptic ACh availability [37].

5. Vascular Dysfunction

Cerebrovascular pathology is increasingly recognized as a major contributor to cognitive impairment and dementia [40]. Chronic cerebral hypoperfusion, resulting from small vessel disease or atherosclerosis, leads to reduced oxygen and glucose delivery, impairing neuronal metabolism and function [40]. Endothelial dysfunction and breakdown of the blood–brain barrier (BBB) further exacerbate neuronal injury by allowing the entry of neurotoxic substances and inflammatory mediators into the brain parenchyma [41]. In addition, impaired vascular clearance mechanisms reduce the removal of amyloid-β, promoting its accumulation in both brain parenchyma and cerebral vessels (cerebral amyloid angiopathy) [41]. Vascular dysfunction also contributes to white matter lesions, microinfarcts, and synaptic disconnection, all of which are associated with cognitive decline [40]. The frequent coexistence of vascular pathology with AD highlights the concept of mixed dementia, emphasizing the importance of vascular health in maintaining cognitive function [40,41].

### 1.3. Modern Medicine and Guideline for Cognitive Impairment Treatment

Current conventional pharmacological interventions for AD primarily include acetylcholinesterase inhibitors (ChEIs; donepezil, galantamine, rivastigmine) and N-methyl-D-aspartate (NMDA) receptor antagonists (memantine). Although these agents can temporarily slow cognitive decline, they do not provide a definitive cure and are often associated with moderate to severe adverse effects. ChEIs is considered first-line therapy for patients with mild to moderate AD, while memantine is recommended for moderate to severe stages, either as monotherapy or in combination with ChEIs [42,43,44]. ChEIs exert their therapeutic effects by inhibiting acetylcholinesterase, thereby increasing acetylcholine levels and enhancing cholinergic neurotransmission in the brain. However, this mechanism also contributes to a range of adverse effects, particularly within the gastrointestinal system, including nausea, vomiting, abdominal pain, anorexia, and diarrhea, which may lead to treatment discontinuation. Additional side effects include muscle weakness, urinary incontinence, dizziness, headache, bradycardia, extrapyramidal symptoms, and sleep disturbances. Similarly, memantine, which modulates glutamatergic neurotransmission through NMDA receptor antagonism, may cause dizziness, constipation, and, in some cases, severe hypotension leading to falls [43,44]. Given comparable efficacy among ChEIs, treatment selection is typically based on drug properties and patient-specific factors, with gradual dose titration recommended to improve tolerability. Despite these therapeutic options, their limited efficacy and tolerability, along with disease progression, often result in treatment discontinuation, particularly in advanced stages where clinical benefits diminish. These limitations have prompted increasing interest in alternative and complementary approaches for the prevention and management of cognitive impairment. In this context, herbal medicines and phytochemicals have gained considerable attention as potential neuroprotective agents. Traditional medicinal systems have long utilized botanical extracts such as *Ginkgo biloba*, *Bacopa monnieri*, and *Panax ginseng* to enhance memory and cognitive function. These natural products are proposed to exert their effects through multiple molecular pathways, including PI3K/Akt, Nrf2/HO-1, NF-κB, and BDNF/TrkB signaling, thereby reducing oxidative stress, suppressing neuroinflammation, promoting neuroplasticity, and modulating neurotransmitter systems [5].

Importantly, owing to their complex phytochemical composition, herbal medicines may exert multi-target pharmacological effects across several interconnected pathways, potentially offering a complementary pharmacological strategy to conventional single-target approaches [45,46]. Therefore, herbal medicine represents a promising complementary strategy, particularly for exploring multimodal neuroprotective mechanisms alongside standard care or when conventional therapies provide limited efficacy.

## 2. Materials and Methods

This narrative review was based on a comprehensive evaluation of the available literature concerning herbal medicines, standardized botanical extracts, isolated phytochemicals, and traditional herbal formulations for cognitive impairment. Relevant publications available up to July 2026 were identified through searches of major scientific databases, including PubMed, Scopus, Web of Science, ScienceDirect, and Google Scholar. The literature search followed an iterative, keyword-based narrative review approach rather than a prespecified systematic-search protocol. Searches were conducted between January and July 2026 and included publications available up to July 2026. The search strategy combined terms related to cognitive disorders and herbal interventions, including “cognitive impairment,” “mild cognitive impairment,” “memory impairment,” “dementia,” “Alzheimer’s disease,” “herbal medicine,” “medicinal plant,” “plant extract,” “phytochemical,” “traditional herbal formulation,” “neuroprotection,” “cholinesterase inhibition,” “amyloid-beta,” “tau phosphorylation,” “oxidative stress,” “neuroinflammation,” and “clinical trial.” Search terms and combinations were adapted iteratively according to database functionality and the specific topic being examined. Additional relevant publications were identified by screening the reference lists of retrieved articles, systematic reviews, and meta-analyses. Retrieved publications were initially screened for relevance based on titles and abstracts. Potentially eligible studies were subsequently assessed in full text when necessary. Eligibility decisions were based on the predefined scope and inclusion/exclusion criteria of the review. Because this review was designed as a narrative rather than a systematic review, complete database-specific Boolean search strings and numerical records for database retrieval, duplicate removal, title/abstract screening, full-text assessment, and exclusions were not prospectively archived. Therefore, these data were not retrospectively reconstructed, and a formal PRISMA-style study-selection flow diagram was not prepared.

Evidence was extracted from in vitro, in vivo, and clinical studies investigating the effects of herbal interventions on cognitive function and the pathological mechanisms associated with cognitive decline. In vitro evidence included enzyme-based assays, neuronal injury models, amyloid-related models, neuroinflammatory models, and studies of neuronal differentiation or synaptic plasticity. In vivo studies were considered when they evaluated behavioral, biochemical, molecular, or histopathological outcomes in established models of cognitive impairment. Clinical evidences included randomized controlled trials, controlled clinical studies, systematic reviews, and meta-analyses involving healthy adults with memory complaints, older adults, patients with mild cognitive impairment, and individuals with AD or related cognitive disorders. Information was synthesized according to the type of intervention, experimental model, cognitive outcomes, proposed mechanisms, and reported safety. For clinical studies, data extraction additionally included sample size, intervention dose, treatment duration, quantitative effect estimates where reported, and adverse events or tolerability findings. Because substantial heterogeneity existed across herbal preparations, study populations, outcome measures, and trial designs, no formal meta-analysis was performed; quantitative findings were therefore summarized descriptively and presented as reported in the original studies. For clinically important interventions, additional data were extracted where available, including comparator, primary endpoint, quantitative effect estimate, 95% confidence interval, statistical significance, adverse events, and major risk-of-bias considerations. Statistical significance was not considered synonymous with clinical relevance; effect magnitude, precision, study design, and consistency with other clinical evidence were considered when interpreting treatment effects. Because of the narrative-review design and the heterogeneity of the included study types, no formal risk-of-bias instrument was applied across all studies. Methodological quality was instead considered qualitatively during evidence interpretation, including study design, randomization and blinding, adequacy of control groups, sample size, intervention standardization, treatment duration, attrition, outcome reporting, and completeness of safety reporting.

Where reported in the original studies, additional information relevant to botanical authentication and chemical standardization was also extracted, including botanical authentication procedures, voucher specimen information, plant part, geographical origin, extraction method, extraction ratio or yield, marker compounds, chemical fingerprints, extract standardization, and batch information. These variables were incorporated into the evidence tables where available, while information not reported in the original publication was designated as “NR” (not reported). Particular attention was given to cholinergic modulation, antioxidant and mitochondrial protection, anti-neuroinflammatory activity, amyloid-β- and tau-related pathways, neurotrophic signaling, and synaptic plasticity. Studies were excluded from the main synthesis if essential information on the intervention, study model or population, dose or concentration, treatment duration, comparator where applicable, or cognition-related outcomes was not reported. Botanical identity was considered unclear when the intervention could not be reliably assigned to a stated plant species or botanical source. Studies were considered unrelated to cognition when they did not assess cognitive performance, validated cognitive outcomes, or mechanisms directly relevant to cognitive impairment. Eligibility was determined from the full-text report using these criteria consistently across studies. The collected evidence was critically evaluated and narratively integrated to identify therapeutic potential, safety concerns, methodological limitations, and priorities for future research. For interpretation, the evidence was considered according to an explicit hierarchy: in vitro mechanistic evidence, in vivo animal evidence, early or exploratory clinical evidence, randomized controlled clinical evidence, systematic reviews and meta-analyses, and, finally, the level of evidence required to support clinical recommendations. Findings from lower levels of evidence were not considered interchangeable with, or directly predictive of, clinical efficacy. As an inherent limitation of the narrative-review design, study identification and selection were not based on a fully prespecified systematic protocol, which may introduce literature-selection bias and limit the reproducibility and comprehensiveness of the evidence synthesis. Accordingly, the findings should be interpreted as a critical narrative synthesis rather than an exhaustive systematic evaluation of all available evidence.

## 3. Results

### 3.1. Preclinical Evidence of Herbal Medicines for Cognitive Impairment

#### 3.1.1. In Vitro Evidence

In vitro studies have provided important preliminary mechanistic insight evidence supporting the neuroprotective potential of herbal medicines and phytochemical-rich preparations against key pathological processes associated with cognitive impairment and AD. As summarized in Table 1, the reported experimental models can be broadly categorized into enzyme-based assays, neuronal injury models, amyloid-β-related models, neuroinflammatory models, and neuronal differentiation or synaptic plasticity models. These systems collectively reflect major mechanistic targets involved in cognitive decline, including cholinergic dysfunction, oxidative stress, excitotoxicity, amyloidogenesis, tau pathology, mitochondrial impairment, neuroinflammation, and impaired neuronal plasticity. A considerable proportion of the studies focused on cholinesterase inhibition, particularly acetylcholinesterase (AChE) and butyrylcholinesterase (BChE), which are well-established pharmacological targets for symptomatic management of AD. Several plant extracts showed promising inhibitory effects, including *Aframomum melegueta* [47], *Annona crassiflora* [48], *Evolvulus alsinoides* [49], *Limonium spathulatum* [50], *Mangifera indica* [51], *Plantago lanceolata* [52], *Punica granatum* [53], *Reseda alba* [54], *Terminalia chebula* [55], *Teucrium polium* [56], and *Tinospora cordifolia* [57]. Notably, some extracts demonstrated inhibitory activity within a similar experimental range to reference compounds such as galantamine, donepezil, rivastigmine, or tacrine under specific in vitro assay conditions, particularly against BChE [43,46,48,51,52]. However, these isolated enzymatic findings must not be interpreted as evidence of therapeutic equivalence, as comparable in vitro potency does not reflect clinical efficacy, pharmacokinetic profiles, central nervous system penetration, or safety in humans. These findings demonstrate plausible biochemical interactions that warrant further investigation regarding their ability to modulate cholinergic neurotransmission. However, the potency varied markedly depending on plant species, solvent fraction, and target enzyme, indicating the importance of phytochemical composition and extraction method in determining bioactivity. Beyond cholinesterase inhibition, many herbal extracts exhibited cytoprotective effects in neuronal cell models challenged with oxidative or excitotoxic insults. For example, *Abelmoschus manihot* [58], Aquilariae Lignum [59], *Arctium lappa* [60], *Bacopa monnieri* [61], *Tinospora cordifolia* [62], and *Trachyspermum ammi* [63] protected PC12, HT22, or primary neuronal cells against H_2_O_2_-, glutamate-, or amyloid-β-induced toxicity. These protective effects were commonly associated with reduced reactive oxygen species production, preservation of mitochondrial membrane potential, attenuation of apoptotic cell death, and improvement in neuronal survival [58,60,62,63,64]. While such cellular models do not demonstrate therapeutic efficacy in vivo, these observations provide valuable hypothesis-generating evidence, particularly relevant because oxidative stress and mitochondrial dysfunction are central contributors to neuronal vulnerability during cognitive impairment and neurodegenerative progression.

Several studies also addressed amyloid-β-related mechanisms, which are highly relevant to AD pathology. Extracts or compounds from *Centella asiatica* [65], *Ginkgo biloba* [66], *Metasequoia glyptostroboides* [67], *Panax ginseng* [68], and *Terminalia chebula* [69] reduced amyloid-β-induced neurotoxicity, inhibited amyloid aggregation, promoted disaggregation of preformed fibrils, or shifted amyloid precursor protein processing toward a non-amyloidogenic pathway [65,66,67,69,70]. In addition, trans-crocin 4 and trans-crocetin from *Crocus sativus* modulated both amyloidogenic and tau-related pathways by reducing β- and γ-secretase expression and suppressing tau phosphorylation [71]. These findings indicate that some herbal-derived compounds exhibit modulatory actions on pathways implicated in disease progression in simplified assay systems, rather than only providing symptomatic cholinergic enhancement. Neuroinflammation was another important mechanistic theme identified in the in vitro evidence. Extracts from *Ardisia elliptica* and *Withania somnifera* attenuated inflammatory responses in LPS-stimulated BV2 microglial cells, RAW264.7 macrophages, or primary microglial cells by suppressing proinflammatory mediators and oxidative stress [72,73]. The anti-inflammatory effects of Ardisia elliptica were additionally linked to inhibition of JNK, ERK1/2, p38, and NF-κB signaling pathways [72]. Since chronic microglial activation and neuroinflammation contribute to neuronal damage and cognitive decline, these findings support the potential of anti-inflammatory herbal medicines as preclinical candidates for further in vivo evaluation. Interestingly, some herbal preparations also promoted neuronal differentiation, neurite outgrowth, synaptogenesis, or neurotrophic signaling that may support neural plasticity. *Bacopa monnieri* enhanced neurite outgrowth and upregulated brain-derived neurotrophic factor expression in amyloid-β-treated differentiated PC12 cells [64], whereas *Lithocarpus litseifolius* promoted neuronal differentiation and maturation in Neuro-2a cells and neural stem/progenitor cells [74]. Similarly, *Ziziphus jujuba* induced neuronal differentiation through increased expression of neuronal cytoskeleton proteins [75], while *Undaria pinnatifida* promoted spinogenesis, synaptogenesis, and presynaptic plasticity in hippocampal neurons [76]. Collectively, these in vitro findings provide exploratory mechanistic evidence that certain botanical extracts can influence pathways relevant to neuronal integrity and synaptic plasticity; however, rigorous in vivo and clinical studies remain essential to confirm whether these cellular actions translate into functional cognitive benefits.

**Table 1:** In vitro evidence of herbal medicines and phytochemical-rich preparations for cognitive impairment.

Plants	Botanical Authentication and Chemical Standardization	Experimental Design/Model	Bioactive Extract/Compound	Effective Dose	Key Findings
*Abelmoschus manihot* (L.) Medik [58]	Flower water extract; authentication/voucher and marker standardization NR.	H_2_O_2_-treated PC12 cells	Water extract of flowers	125 and 500 μg/mL	Extracts showed dose-dependent protective effect on PC12 cells against H_2_O_2_-induced cell death, ROS production, and inflammation.
*Aframomum melegueta* K.Schum. [47]	Seed fractions; kaempferol-enriched active fraction characterized; voucher/standardization NR	Cholinesterase activity assay	n-Hexane or Ethyl acetate fraction of seeds	16.83 and 11.67 μg/mL (IC50 for AChE) 7.54 and 5.21 μg/mL (IC50 for BChE)	Both extracts inhibited AChE (IC50 = 16.83 and 11.67 μg/mL for the n-hexane and ethyl acetate fractions, respectively) and BChE (IC50 = 7.54 and 5.21 μg/mL, respectively). Rivastigmine was included as the reference inhibitor under the same assay conditions.
*Annona crassiflora* Mart. [48]	Authenticated material; voucher HUFU68467; alkaloid fraction characterized	Cholinesterase activity assay	Alkaloidal fraction of fruit peels	57.7 ± 9.0 μg/mL (IC50 for BChE) 57.0 ± 9.7 μg/mL (IC50 for AChE)	The alkaloidal fraction inhibited both BChE (IC50 = 57.7 ± 9.0 μg/mL) and AChE (IC50 = 57.0 ± 9.7 μg/mL). Galantamine was included as the reference inhibitor under the same assay conditions.
*Aquilariae Lignum* [59]	Heartwood ethanol extract; authentication/voucher and marker standardization NR	Glutamate-treated HT22 hippocampal neuronal cells	Ethanol extract of heartwoods	5–25 μg/mL	Extract showed dose-dependent protective effect on neuronal cells against glutamate-induced apoptotic cell death and ROS production.
*Arctium lappa* L. [60]	Root ethyl acetate extract; authentication/voucher and marker standardization NR	Glutamate-treated PC12 cells	Ethyl acetate extract of roots	40 and 80 μg/mL	Extracts showed dose-dependent protective effect on PC12 cells against glutamate-induced cell death, ROS production, and mitochondrial membrane potential disruption.
*Ardisia elliptica* Thunb. [72]	Fruit hydroalcoholic extracts; phytochemical characterization reported; formal standardization NR	LPS-treated BV2 or RAW264.7 cells	50% or 90% Hydroalcoholic extracts of fruits	10 μg/mL or 5 μg/mL	Both extracts showed protective effect on BV2 and RAW264.7 cells against LPS-induced inflammation by attenuating expression of proinflammatory mediators. In addition, they inhibited amyloid-β fibril formation and promoted disassembled preformed fibril aggregates in BV2 cells.
*Bacopa monnieri* (L.) Wettst. [61]	Leaf hexane extract; quantitative marker standardization NR	Glutamate-treated HT22 hippocampal neuronal cells	Hexane extract of leaves	10 µg/mL	Extract showed protective effect on neuronal cells against glutamate-induced cell death, ROS production, mitochondrial stress, and ER stress
*Bacopa monnieri* (L.) Wettst. [64]	Leaf hexane extract; quantitative marker standardization NR	Amyloid-β-treated differentiated PC12 neuronal cells	Hexane extract of leaves	10 µg/mL	Extract showed dose-dependent protective effect on neuronal cells against amyloid-β-induced cell death, potentiated neurite outgrowth, and upregulated BDNF expression
*Centella asiatica* (L.) Urb. [65]	Whole-plant ethanol extract; authentication/voucher and marker standardization NR	Amyloid-β-treated PC12 or IMR32 neuronal cells	Ethanol extract of whole plant	25–100 μg/mL	Extract showed protective effect on neuronal cells against amyloid-β-induced cell death and ROS production.
*Crocus sativus* L. [71]	trans-Crocin 4 and trans-crocetin isolated and structurally characterized	SH-SY5Y neuronal cells overexpressing APP (SH-SY5Y-APP) and PC12 expressing hyper-phosphorylated tau (PC12-htau)	*trans*-Crocin 4 or *trans*-Crocetin	1 mM or 10 µM	Compound treatment blocks the amyloidogenic pathway in neuronal cells by reducing the expression of ß- and γ-secretases, key enzymes of the amyloidogenic pathway, as well as suppressing tau phosphorylation pathway.
*Crocus sativus* L. [77]	Stigma methanol extract; chemical characterization reported; formal standardization NR	Cholinesterase activity assay	Methanolic extract of stigmas	19.76 ± 1.20 μg/mL (IC50 for BChE) 131.82 ± 3.03 μg/mL (IC50 for AChE)	The methanolic stigma extract inhibited both BChE (IC50 = 19.76 ± 1.20 μg/mL) and AChE (IC50 = 131.82 ± 3.03 μg/mL). Galantamine was included as the reference inhibitor under the same assay conditions.
*Elaeagnus glabra* f. *oxyphylla* (Servett.) W. T. Lee [78]	Branch ethanol extract; active flavonoid constituents characterized; formal standardization NR	Amyloid ß aggregation assay	Ethanol extract of branches	32.01 μg/mL (IC50 for amyloid ß)	Extract demonstrated dose-dependent inhibitory effect on amyloid ß aggregation (IC50 = 32.01 μg/mL), which its effect at 100 μg/mL is comparable to that of a known inhibitor, morin.
*Evolvulus alsionoides* L. [49]	Authenticated leaves; multi-solvent extracts; quantitative phenolic characterization	Cholinesterase activity assay	Methanol or water extracts of leaves	4.77 ± 0.04 and 4.46 ± 0.03 μg/mL (IC50 for AChE)	Methanolic and aqueous leaf extracts inhibited AChE, with IC50 values of 4.77 ± 0.04 and 4.46 ± 0.03 μg/mL, respectively. Galantamine was included as the reference inhibitor under the same assay conditions.
*Ginkgo biloba* L. [66]	Standardized leaf extract EGb 761 (Tanakan^®^)	Amyloid ß-treated hippocampal primary cells or mixed glial/hippocampal neuronal cells	Extract of leaves (Tanakan^®^ EGb761)	50–100 μg/mL	Extract showed dose-dependent protective effect on neuronal cells against amyloid ß-induced cell death, as well as intracellular and mitochondrial ROS production.
*Ginkgo biloba* L. [70]	Ginkgolide B as a defined compound; extract standardization not applicable	Amyloid ß-treated primary hippocampal neuron cells	Ginkgolide B	40 μg/mL	Compound showed a protective effect on hippocampal neuron cells against amyloid β-induced apoptosis by enhancing the expression of BDNF.
*Huperzia serrata* (Thunb.) Trevis [79]	Authenticated; voucher NSP01-01; standardized NSP01 extract with UHPLC-MS profile	Glutamate- or amyloid ß-treated primary neuronal cells	Microwave-assisted water extract (NSP01) of whole plant	0.5–33.3 μg/mL (for glutamate) 0.0025–0.5 μg/mL (for amyloid ß) 0.0042–42 μg/mL (for AChE)	Extract showed dose-dependent protective effect on neuronal cells against glutamate-induced cell death and neurite network reduction, whereas only the protective effect on cell survival but not neurite network was observed in amyloid ß-treated model. Extract also demonstrated inhibitory activity against AChE in a dose-dependent manner.
*Limonium spathulatum* (Desf.) Kuntze [50]	Leaf extracts; phytochemical profiling reported; marker standardization NR	Cholinesterase activity assay	Ethanol or water or hydroethanolic extracts of leaves	0.27 ± 0.09, 0.06 ± 0.02, and 0.03 ± 0.01 mg/mL (IC50 for BChE) 1.75 ± 0.06, 0.23 ± 0.04, and 0.31 ± 0.05 mg/mL (IC50 for AChE)	Ethanolic, aqueous, and hydroethanolic leaf extracts inhibited BChE (IC50 = 0.27 ± 0.09, 0.06 ± 0.02, and 0.03 ± 0.01 mg/mL, respectively) and AChE (IC50 = 1.75 ± 0.06, 0.23 ± 0.04, and 0.31 ± 0.05 mg/mL, respectively). Galantamine was included as the reference inhibitor under the same assay conditions.
*Lithocarpus litseifolius* (Hance) Chun [74]	Whole-plant hydroalcoholic extract; quantitative marker standardization NR	Neuro-2a or neural stem/progenitor cells	Hydroalcoholic extract of whole plant	5–20 μg/mL	Extract demonstrated dose-dependent effect on promoting differentiation and maturation of neuronal cells through enhancing neurite outgrowth and inducing the expressions of mature neuronal cell-specific proteins.
*Mangifera indica* L. [51]	Leaf ethanol extract; LC-MS/MS phenolic characterization; formal standardization NR	Cholinesterase activity assay	Ethanol extract of leaves	16.116 μg/mL (IC50 for AChE) 8.556 μg/mL (IC50 for BChE)	Extract demonstrated inhibitory activity against AChE (IC50 = 16.116 μg/mL vs. 3.19 μg/mL) and BChE (IC50 = 8.556 μg/mL vs. 2.54 μg/mL) compared to tacrine.
*Metasequoia glyptostroboides* Hu & W.C.Cheng [67]	Defined isolated diterpenoids; extract standardization not applicable	Amyloid ß-treated PC12 cells	Sandaracopimarinol or sandaracopimaradien-19-ol	20 and 100 μg/mL	Both compounds showed dose-dependent protective effect on PC12 cells against amyloid ß-induced toxicity by reducing the aggregation and enhancing the disaggregation of pre-formed amyloid ß aggregates.
*Panax ginseng* C.A. Meyer [80]	Root essential oil; quantitative marker standardization NR	β-secretase and cholinesterase activity assays	Essential oil from roots	250 μg/mL	Essential oil showed strong inhibition toward β-secretase (41.4%), AChE (77.4%), and BChE (94.1%), which are key enzymes related to AD.
*Panax ginseng* C.A. Meyer [68]	Red ginseng oil; chemical composition characterized	Amyloid ß-treated PC12 neuronal cells	Essential oil from roots	10–100 μg/mL	Essential oil showed dose-dependent protective effect on neuronal cells against amyloid β-induced apoptotic cell death, ROS production, mitochondria dysfunction, and inflammatory responses.
*Panax ginseng* C.A. Meyer [81]	Ginsenoside Rg1 as a defined compound	Amyloid ß-treated PC12 neuronal cells	Ginsenoside Rg1	10 μM	Compound showed protective effect on neuronal cells against amyloid β-induced cell death and ROS production, enhanced mitochondrial bioenergetics, and restored mitochondrial damage.
*Plantago lanceolata* L. [52]	Leaf ethanol extract; phytochemical composition characterized; formal standardization NR	Cholinesterase activity assay	Ethanol extract of leaves	8.77 ± 0.52 μg/mL (IC50 for AChE) 7.37 ± 0.53 μg/mL (IC50 for BChE)	The ethanolic leaf extract inhibited AChE (IC50 = 8.77 ± 0.52 μg/mL) and BChE (IC50 = 7.37 ± 0.53 μg/mL). Tacrine was included as the reference inhibitor under the same assay conditions.
*Punica granatum* L. [53]	Leaf extracts profiled by UPLC-MS/MS; ellagic acid predominant	Cholinesterase activity assay	Ethanol or Methanol extract of leaves	0.0017 μg/mL (IC50 for AChE)	The leaf extracts showed AChE inhibitory activity, with an IC50 of 0.0017 μg/mL under the reported assay conditions. Tacrine was included as the reference inhibitor.
*Reseda alba* L. [54]	Authenticated; voucher deposited; phenolic profile characterized	Cholinesterase activity assay	Dichloromethane or ethyl acetate or n-butanol extracts of whole plant	43.01 ± 0.28, 13.68 ± 0.84, and 31.43 ± 8.26 μg/mL (IC50 for BChE) 51.52 ± 1.37, 61.02 ± 0.98, and 48.2 ± 0.28 μg/mL (IC50 for AChE)	The dichloromethane, ethyl acetate, and n-butanol extracts inhibited BChE (IC50 = 43.01 ± 0.28, 13.68 ± 0.84, and 31.43 ± 8.26 μg/mL, respectively) and AChE (IC50 = 51.52 ± 1.37, 61.02 ± 0.98, and 48.2 ± 0.28 μg/mL, respectively). Galantamine was included as the reference inhibitor under the same assay conditions.
*Rumex hastatus* D. Don [82]	Whole-plant crude flavonoid extract; phytochemical characterization reported; formal standardization NR	Cholinesterase activity assay	Crude flavonoid extract of whole plant	20 μg/mL (IC50 for AChE) 175 μg/mL (IC50 for BChE)	The crude flavonoid extract inhibited AChE (IC50 = 20 μg/mL) and BChE (IC50 = 175 μg/mL). Galantamine was included as the reference inhibitor under the same assay conditions.
*Terminalia chebula* Retz. [69]	Fruit water extract; quantitative marker standardization NR	APPswe transgenic SH-SY5Y cells	Water extract of fruits	40 μg/mL	Extract showed a protective effect APPswe-transgenic SH-SY5Y cells by blocking amyloid β generation and enhancing of amyloid β hydrolysis and autophagy.
*Terminalia chebula* Retz. [55]	Fruit ethyl acetate extract; phytochemical characterization reported; formal standardization NR	Cholinesterase activity assay	Ethyl acetate extract of fruits	52.32 ± 0.04 μg/mL (IC50 for BChE) 45.44 ± 0.03 μg/mL (IC50 for AChE)	The ethyl acetate fruit extract inhibited BChE (IC50 = 52.32 ± 0.04 μg/mL) and AChE (IC50 = 45.44 ± 0.03 μg/mL). Donepezil was included as the reference inhibitor under the same assay conditions.
*Teucrium polium* L. [56]	Aerial-part hydroalcoholic extract; phytochemical characterization reported; formal standardization NR	Cholinesterase activity assay	Hydroalcoholic extract of aerial part	4.93 μg/mL (IC50 for AChE) 28.69 μg/mL (IC50 for BChE)	The hydroalcoholic aerial-part extract showed dose-dependent inhibition of AChE (IC50 = 4.93 μg/mL) and BChE (IC50 = 28.69 μg/mL). Galantamine was included as the reference inhibitor under the same assay conditions.
*Tinospora cordifolia* (Willd.) Hook.f. & Thomson [57]	Tinosporide and 8-hydroxytinosporide as defined compounds	Cholinesterase activity assay	Tinosporide or 8-hydroxytinosporide	13.45 ± 0.144 and 46.71 ± 0.511 μg/mL (IC50 for AChE) 408.50 ± 17.197 and 317.26 ± 6.918 μg/mL (IC50 for BChE)	Both compounds demonstrated inhibitory activity against AChE (IC50 = 13.45 ± 0.144 and 46.71 ± 0.511 μg/mL vs. 6.31 ± 0.089 μg/mL), compared to donepezil. Their inhibitory activity against BChE was lower than that of donepezil (IC50 = 408.50 ± 17.197 and 317.26 ± 6.918 μg/mL vs. 11.93 ± 0.129 μg/mL).
*Tinospora cordifolia* (Willd.) Hook.f. & Thomson [62]	Stem butanol extract; quantitative marker standardization NR	Glutamate-treated primary cerebellar neurons	Butanol extract of stems	20 μg/mL	Tinosporide and 8-hydroxytinosporide inhibited AChE (IC50 = 13.45 ± 0.144 and 46.71 ± 0.511 μg/mL, respectively) and BChE (IC50 = 408.50 ± 17.197 and 317.26 ± 6.918 μg/mL, respectively). Donepezil was included as the reference inhibitor under the same assay conditions.
*Trachyspermum ammi* (L.) Sprague [63]	Seed hexane/ethyl acetate extracts; phytochemical characterization reported; formal standardization NR	H_2_O_2_-treated HT22 hippocampal neuronal cells	Hexane or ethyl acetate extracts of seed	50 μg/mL	Both extracts showed a protective effect on HT22 hippocampal neuronal cells against H_2_O_2_-induced cell death, ROS production and mitochondrial membrane potential disruption.
*Undaria pinnatifida* [76]	Ethanol extract; quantitative marker standardization NR	Hippocampal Neurons	Ethanol extract of seaweeds	5 μg/mL	Extract demonstrated the ability to promote spinogenesis and synaptogenesis by increasing the number of dendritic filopodia and spines, enhancing the formation of excitatory synapses, and potentiating synaptic transmission through enlargement of reserve vesicle pools at presynaptic terminals.
*Withania somnifera* (L.) Dunal [73]	Aqueous leaf extract; withaferin A and withanone detected	LPS-treated BV2 or primary microglial cells	Water extract of leaves	0.2%	Extract showed protective effect on microglial cells against LPS-induced inflammation by attenuating production of ROS and expression of proinflammatory mediators.
*Ziziphus jujuba* Mill. [75]	Standardized fruit extract used	PC12 neuronal cells	Water extract of fruits	2 mg/mL	Extract demonstrated the effect on neuronal differentiation though promoting neurite outgrowth and inducing the expressions of neuronal cell-specific cytoskeleton proteins or neurofilaments by 100–150% in a dose-dependent manner.

AChE, Acetylcholinesterase; APPswe, Amyloid precursor protein Swedish mutation; BChE, Butyrylcholinesterase; BDNF, Brain-derived neurotrophic factor; ER, Endoplasmic reticulum; H_2_O_2_, Hydrogen peroxide; IC50, Half-maximal inhibitory concentration; LPS, Lipopolysaccharide; ROS, Reactive oxygen species. NR, not reported in the original study. For isolated compounds, extract-level standardization was considered not applicable. IC50 values reported across different studies should be interpreted with caution because enzyme sources, substrates, solvent systems, incubation conditions, assay formats, and detection methods may differ. Accordingly, inhibitory potency should not be ranked across studies unless experimental conditions are directly comparable. Comparisons with reference inhibitors in this table refer only to those performed within the respective study under the reported assay conditions.

#### 3.1.2. In Vivo Evidence

Preclinical in vivo studies provide important mechanistic support for the potential application of herbal medicines in cognitive impairment, particularly by demonstrating improvements in behavioral memory tasks together with changes in biochemical and molecular markers related to neurodegeneration [83,84,85]. Most available studies have used pharmacologically induced models, especially scopolamine-induced amnesia, which mimics transient muscarinic cholinergic dysfunction and produces deficits in learning, memory acquisition, spatial memory, and recognition memory [83,86,87]. Other disease-relevant models, such as intracerebroventricular streptozotocin-induced sporadic AD-like models and chemically induced dementia models using aluminum chloride/D-galactose or L-methionine, have also been used to evaluate herbal extracts and phytochemicals under conditions involving oxidative stress, neuroinflammation, amyloidogenic changes, and impaired neuronal signaling [88]. Behavioral endpoints commonly include the Morris water maze, Y-maze, elevated plus maze, passive avoidance test, radial arm maze, and novel object recognition test, allowing assessment of spatial learning, working memory, retention memory, and recognition memory across different experimental paradigms [86,89]. Across these models, many herbal extracts improved cognitive performance in parallel with reductions in acetylcholinesterase activity, restoration of acetylcholine-related signaling, attenuation of oxidative stress, and protection of hippocampal or cortical neuronal integrity [53,90]. These findings are consistent with the multifactorial pathophysiology of cognitive impairment, in which cholinergic deficits, oxidative damage, neuroinflammation, synaptic dysfunction, amyloid/tau-related pathology, and neuronal loss contribute simultaneously to memory decline [85,91].

Several studies in the current evidence base were not included in the main herbal medicine table because their interventions were not clearly classified as single herbal extracts or traditional multi-herbal formulations. Instead, these studies investigated isolated phytochemicals, complex antioxidant products, or dietary botanical mixtures. For example, p-coumaric acid, a naturally occurring phenolic acid found in various medicinal plants and food sources, demonstrated cognitive-protective effects in aluminum chloride/D-galactose- and L-methionine-induced dementia models. These effects were associated with restoration of endogenous antioxidant defenses and reductions in acetylcholinesterase activity, thiobarbituric acid-reactive substances, nitrite levels, and tumor necrosis factor-α, suggesting that its neuroprotective activity may involve cholinergic modulation, attenuation of oxidative stress, and suppression of neuroinflammatory responses [88]. γ-Mangostin, a xanthone from *Garcinia mangostana*, showed both in vitro neuroprotection against oxidative stress and in vivo improvement of scopolamine-induced memory impairment [92]. Formononetin isolated from *Sophora secundiflora* also demonstrated neuroprotective activity in a scopolamine model, with modulation of AChE, acetylcholine, GSH, MDA, dopamine, and noradrenaline [93]. In addition, Antia, a complex natural antioxidant product derived from mushroom and plant sources, improved streptozotocin-induced sporadic AD-like cognitive dysfunction by targeting amyloidogenic, inflammatory, oxidative stress, autophagy, and JAK2/STAT3-related pathways [94]. These studies are valuable for mechanistic interpretation but are better discussed narratively rather than grouped together with whole herbal medicines or traditional formulations.

The main vivo evidence for herbal medicines is summarized in Table 2. The table focuses on whole herbal extracts, standardized herbal preparations, and traditional multi-component formulations. For clarity, isolated phytochemicals and complex non-formula products are excluded from the table and discussed separately above.

These in vivo studies indicate that herbal medicines may improve cognitive impairment through overlapping mechanisms involving cholinergic restoration, antioxidant defense, anti-inflammatory regulation, neurotrophic signaling, and neuronal protection. Nevertheless, most evidence remains preclinical and heterogeneous; therefore, future studies should emphasize standardized extracts, validated marker compounds, pharmacokinetic profiling, brain-distribution studies, long-term toxicity evaluation, and disease-relevant animal models that more closely reflect the chronic progression of MCI and AD. Importantly, the concentrations and doses reported across experimental systems should not be interpreted as equivalent exposures. Concentrations used in biochemical or cellular assays (e.g., μM or μg/mL), doses administered in animal studies (e.g., mg/kg), and oral doses used in clinical studies (e.g., mg/day or g/day) reflect fundamentally different pharmacological contexts and cannot be directly compared or converted without appropriate pharmacokinetic data. Their biological relevance depends on factors including route of administration, absorption, formulation, metabolism, systemic distribution, clearance, and tissue exposure. Accordingly, dose-related findings should be interpreted within the specific experimental system in which they were obtained. Clinical evidence was interpreted separately from preclinical findings and weighted according to study design and methodological rigor. Small or exploratory human studies were considered preliminary, whereas greater evidential weight was assigned to adequately controlled randomized trials and systematic reviews or meta-analyses.

### 3.2. Clinical Evidence of Herbal Medicines and Herbal Formulations

Clinical findings were interpreted according to both statistical and clinical relevance. A statistically significant change in MMSE, MoCA, ADAS-cog, or other cognitive measures was not considered sufficient evidence of clinically meaningful benefit in the absence of an adequate effect magnitude, precision, appropriate comparator, and supportive functional or longitudinal outcomes. Because the included clinical studies involved heterogeneous populations, including healthy participants, individuals with subjective memory complaints, patients with MCI, patients with AD, and other neurocognitive conditions, findings were interpreted within their respective clinical populations. Cognitive effects observed in healthy adults or individuals with subjective complaints were not extrapolated to therapeutic efficacy in MCI or AD, and conclusions were therefore made according to the population studied.

#### 3.2.1. Clinical Trials of Single Herbal Medicines and Extracts

Clinical trials of single herbal medicines and standardized botanical extracts for cognitive impairment have primarily focused on memory-related outcomes, particularly in healthy older adults, individuals with subjective memory complaints, and patients with MCI. As summarized in Table 3, *Panax ginseng* [98], hydroponically cultivated red ginseng HRG80 [99], *Bacopa monnieri*, saffron (*Crocus sativus*) [100], and sage-based preparations represent the major single-herb or extract-based interventions evaluated in randomized clinical studies. Among these, *Panax ginseng* showed clinically relevant benefits in Korean volunteers with MCI, in which 3 g/day of ginseng powder for 24 weeks significantly improved Rey Complex Figure Test immediate recall and 20-min delayed recall compared with placebo, suggesting a potential role in visual memory enhancement [98]. Saffron also demonstrated promising findings in a one-year randomized study of patients with amnestic multidomain MCI, where Mini-Mental State Examination (MMSE) scores improved in the intervention group while the control group deteriorated; supportive MRI, EEG, and ERP findings were also reported. However, this saffron trial was limited by small sample size and single-blind design, indicating that further placebo-controlled trials are required before definitive conclusions can be drawn [100]. Other phytochemical-rich single-herb or extract-based interventions provide supportive but still preliminary evidence. Sage (*Salvia officinalis* and *S. lavandulaefolia*) has been investigated in healthy subjects and patients with cognitive decline, with clinical evidence suggesting possible cognitive-enhancing effects that may be related to cholinesterase inhibition, antioxidant activity, anti-inflammatory properties, and terpenoid- or phenolic-rich phytochemistry. Nevertheless, the systematic review of *Salvia* trials emphasized that interpretation is constrained by methodological limitations, small sample sizes, and variation in herbal preparations [101]. Similarly, combined phytochemical-rich interventions, such as sage–rosemary–Melissa extract, Bacopa–American ginseng–coffee fruit extract, and quercetin-enriched culinary herb formulations, showed improvements in selected outcomes such as delayed recall, working memory, prefrontal cortex activation, ERP components, and cholinesterase or monoamine oxidase activity. However, these studies were generally conducted in healthy adults or specific non-dementia populations and were relatively short in duration [102,103,104].

Beyond individual clinical trials, several systematic reviews and meta-analyses have synthesized the clinical evidence of Chinese herbal medicine (CHM) for mild cognitive impairment (MCI). Overall, these reviews suggest that CHM may provide cognitive benefits in patients with MCI, particularly when assessed using global screening tools such as the MMSE and Montreal Cognitive Assessment (MoCA). A systematic review focusing on MMSE outcomes included 21 studies and reported that most trials favored herbal interventions, either as monotherapy or in combination with other cognition-enhancing treatments. However, the authors also emphasized that methodological flaws limited the reliability of the findings [105]. Similarly, a MoCA-focused systematic review identified 19 randomized controlled trials and suggested that certain orally administered CHM formulations improved MoCA scores by approximately 1.76–2.34 points compared with placebo after 24 weeks of treatment, although the included studies were heterogeneous and generally had methodological weaknesses [106]. More recent evidence provides a cautiously supportive interpretation. A 2022 systematic review of 49 randomized trials found that CHM significantly improved cognitive function measured by MMSE and MoCA and reduced the risk of progression from MCI to dementia compared with no intervention or placebo. Nevertheless, the overall methodological quality of the included trials was relatively low, and the authors concluded that more rigorously designed randomized trials are required to confirm these findings [107]. A 2024 placebo-controlled meta-analysis, which included 13 randomized trials involving 1043 participants, further reported that CHM significantly improved MMSE and MoCA scores compared with placebo. The certainty of evidence was rated as moderate for MMSE and low for MoCA, while adverse events were not significantly different between CHM and placebo; gastrointestinal symptoms were the most commonly reported adverse events [108]. The lower certainty, particularly for MoCA outcomes, reflects concerns regarding study-level risk of bias, heterogeneity and inconsistency across trials, imprecision arising from relatively small studies, indirectness related to variation in formulations and populations, and possible publication bias. Taken together, these findings indicate that CHM may have potential as a complementary intervention for MCI, but the evidence remains limited by heterogeneity of formulas, variable treatment durations, incomplete adverse-event reporting, and risk-of-bias concerns. For AD, systematic evidence is more cautious and supports CHM mainly as an adjunctive rather than stand-alone therapy. A systematic review of CHM for AD found that CHM used in combination with conventional treatment may provide additive cognitive benefits, whereas evidence for CHM monotherapy remains inconclusive. Mechanistically, frequently used herbal constituents and phytochemicals, such as ginsenosides, icariin, catalpol, astragaloside IV, salvianolic acids, and tetramethylpyrazine, have been linked to multi-target actions relevant to AD pathology, including modulation of amyloid-β toxicity, tau hyperphosphorylation, oxidative stress, neuroinflammation, apoptosis, cholinergic dysfunction, and hippocampal neurogenesis. However, safety interpretation remains limited because adverse-event reporting was incomplete in several trials, and some studies reported serious events, including hepatic function impairment and cardiovascular-related events [109]. Therefore, CHM for AD should currently be interpreted as a promising adjunctive therapeutic hypothesis rather than an established monotherapy, and future trials should incorporate standardized herbal products, rigorous placebo control, biomarker-supported diagnosis, and systematic toxicity monitoring.

**Table 3:** Clinical evidence of herbal medicines and phytochemical-rich preparations for cognitive impairment.

Herbal Medicine/Formulation	Intervention Composition, Standardization, and Comparator	Study Design, Population, Sample Size, Dose and Duration	Main Outcomes, Quantitative Effect, and Safety
Brahmi (*Bacopa monnieri*) [110]	BacoMind^®^ *B. monnieri* extract; bacoside A3 > 5% *w*/*w*; 450 mg/day; comparator: placebo	RCT, double-blind; N = 65; memory complaints; 450 mg/day; 12 weeks	Improved delayed recall, memory span, associative and visual memory; effect estimate/AE NR
Brahmi (*Bacopa monnieri*) [111]	MediHerb^®^ *B. monnieri* extract; standardized to 50% bacosides; 300 mg/day; comparator: placebo	RCT, double-blind; N = 54; adults > 65 y; 300 mg/day; 12 weeks	Improved Stroop performance and delayed verbal recall; effect estimate/AE NR
Brahmi (*Bacopa monnieri*) [112]	BacoMind^®^ *B. monnieri* extract; 40–50% bacosides; 300 mg/day; comparator: placebo	RCT, double-blind; N = 98; healthy adults > 55 y; 300 mg/day; 12 weeks	Improved later learning trials and delayed recall; effect estimate/AE NR
Brahmi (*Bacopa monnieri*) [113]	KeenMind^®^ *B. monnieri* extract; 55% bacosides; dose adjusted according to body weight (<90 kg or >90 kg; up to 400 mg/day); comparator: placebo	RCT, double-blind; N = 84; healthy adults; ≤400 mg/day; 12 weeks	Improved delayed paired-word recall; effect estimate/AE NR
Brahmi (*Bacopa monnieri*) [114]	KeenMind^®^ *B. monnieri* extract; standardized to a minimum of 55% bacosides; 300 mg/day; comparator: placebo	RCT, double-blind; N = 46; healthy adults; 300 mg/day; 12 weeks	Improved inspection time and verbal-learning indices; effect estimate/AE NR
Brahmi (*Bacopa monnieri*) [115]	KeenMind^®^ *B. monnieri* extract; standardized to a minimum of 55% bacosides; 300 mg/day; comparator: placebo	RCT, double-blind; N = 107; healthy adults; 300 mg/day; 12 weeks	Fewer false alarms in rapid visual processing; effect estimate/AE NR
Bushen capsule [116]	Bushen capsule, a multi-herb formulation mainly containing Cistanches Herba, Rhizoma Alismatis, and *Polygonum multiflorum*; marker-based chemical standardization NR; comparator: placebo	Placebo-controlled trial; N = 60; aMCI; 2 years	Memory stabilized/improved; fMRI changes; mild decreased appetite, constipation and nausea reported
Davaie Loban [117]	Fixed Traditional Persian Medicine formulation containing *Cyperus rotundus*, *Zingiber officinale*, *Acorus calamus*, *Piper nigrum*, and *Boswellia carterii*; chemical marker standardization NR; comparator: placebo	RCT, double-blind; 44 completers; mild–moderate AD; 12 weeks	ADAS-cog and CDR-SOB significantly improved vs. placebo; detailed effect estimate/AE NR
Di-Tan decoction [118]	Classical Chinese herbal formulation evaluated as an add-on intervention; detailed chemical standardization NR in the study protocol; comparator: placebo plus conventional treatment	RCT protocol; mild–moderate AD; planned 24 weeks	No efficacy data; primary endpoint: ≥4-point ADAS-cog improvement
Ginseng (*Panax ginseng*) [98]	Panax ginseng powder containing 53 mg/g ginsenosides; 3 g/day; comparator: placebo	RCT, double-blind; N = 90; MCI; 3 g/day; 24 weeks	Improved Rey Complex Figure immediate and 20-min delayed recall; AE NR
Hydroponically cultivated red ginseng HRG80 [99]	Hydroponically cultivated red ginseng preparation (HRG80) characterized by ginsenosides and processing-derived rare ginsenosides; quantitative marker standardization NR; comparator as reported in the original trial	Pilot crossover RCT; N = 50; healthy adults	Improved attention, memory and perceived stress; dose/effect estimate/AE NR
Jieduhuayu granules [119]	Chinese herbal granule formulation; detailed botanical composition and chemical marker standardization NR in the reviewed study; comparator: NR	Non-blinded RCT; N = 80; minimal hepatic encephalopathy; 15 days	Improved NCT-A, MMSE and P300; effect estimate/AE NR
Jujadokseo-hwan (JDH) [120]	Multi-herb formulation containing Poria Sclerotium cum Pini Radix, Polygalae Radix, Ginseng Radix, Citri Unshius Pericarpium, Acori Graminei Rhizoma, Angelicae Gigantis Radix, and Glycyrrhizae Radix et Rhizoma; chemical marker standardization NR; comparator: placebo	RCT-based economic analysis; N = 64; MCI; 24 weeks	KQOL-AD improved; EQ-5D slight change; not cost-effective; detailed AE NR
Polyphenolic-rich combined supplement [103]	Combination of *Bacopa monnieri* 300 mg, *Panax quinquefolius* 100 mg, and whole coffee fruit extract 100 mg; detailed quantitative marker standardization NR; comparator: placebo	RCT, double-blind; N = 52; healthy middle-aged adults; 28 days	Improved positive affect/delayed recall; increased prefrontal activation; AE NR
Qinggongshoutao (QGST) [121]	Traditional Chinese herbal pill containing *Cistanche deserticola*, *Rehmannia glutinosa*, *Lycium barbarum*, *Cynomorium songaricum*, *Morinda officinalis*, *Schisandra chinensis*, *Angelica sinensis*, *Dioscorea opposita*, *Achyranthes bidentata*, and *Polygala tenuifolia*; marker-based chemical standardization NR; comparator: placebo/active control as reported	RCT; N = 350; aMCI; 52 weeks	Reduced progression to possible/probable AD; improved ADAS-cog/MMSE; detailed AE NR
Quercetin-enriched culinary herb formulation [104]	Combined extract of *Polygonum odoratum* and *Morus alba* formulated as a quercetin-enriched functional ingredient; quantitative marker specification NR; comparator: placebo	RCT, double-blind; peri/postmenopausal women; 1500 mg/day; 8 weeks	Improved working memory; reduced N100/P300 latency; increased amplitudes; short-term tolerability acceptable
Saffron (*Crocus sativus*) [100]	*Crocus sativus* preparation characterized by crocin, crocetin, safranal, and carotenoid derivatives; quantitative marker standardization NR; comparator: control	Single-blind RCT; amnestic multidomain MCI; 1 year	MMSE improved vs. deterioration in control; MRI/EEG/ERP supportive; AE NR
Sage–rosemary–Melissa combination (SRM) [102]	Combination of *Salvia officinalis*, *Rosmarinus officinalis*, and *Melissa officinalis*, containing phenolic- and terpenoid-rich constituents; quantitative marker standardization NR; comparator: placebo	Pilot RCT; N = 44; healthy subjects; 2 weeks	No overall difference; delayed recall improved only in subgroup < 63 y; AE NR
Shenwu capsule [122]	Defined multi-herb formulation containing *Polygonum multiflorum*, *Panax ginseng*, and *Acorus tatarinowii*; marker-based chemical standardization NR; comparator: donepezil	Phase III RCT; N = 324; aMCI; 24 weeks; vs. donepezil 5 mg/day	ADAS-cog −4.23 points from baseline; delayed recall improved; AEs less frequent than donepezil
Yishen Huazhuo decoction (YHD) [123]	Seven-herb formulation containing Epimedium, Ligustri Lucidi Fructus, Psoralea fruit, Polygoni Multiflori Radix, Astragali Radix, *Ligusticum wallichii*, and *Acorus gramineus*; marker-based chemical standardization NR; comparator: donepezil	Multicenter RCT; N = 144; mild AD; 24 weeks; vs. donepezil	ADAS-cog and MMSE improved in both groups, with a significant time × treatment interaction favoring YHD

NR, not reported in the original study; AE, adverse event. Effect estimates, 95% confidence intervals, statistical significance, and safety findings are presented as reported in the original publications. Key methodological limitations are summarized where evident from the reported study design.

#### 3.2.2. Clinical Trials of Traditional Herbal Formulations

Traditional multi-herb formulations have been evaluated in clinical trials for cognitive impairment, particularly in amnestic mild cognitive impairment (aMCI) and mild-to-moderate AD. In their respective traditional paradigms, these formulas were historically formulated based on holistic theoretical frameworks—such as addressing “kidney essence deficiency,” “phlegm obstruction,” “blood stasis,” or “qi and yin deficiency.” However, these classical diagnostic patterns must be clearly distinguished from experimentally validated biomedical mechanisms; modern pharmacological studies instead hypothesize that any observed clinical effects arise from multi-target actions of co-occurring phytochemicals on defined molecular pathways. Furthermore, because these preparations originate from distinct medical traditions and vary widely in their botanical composition, dosage, preparation methods, and active constituents, each formulation represents an individualized intervention rather than an interchangeable class of therapy. Therefore, clinical outcomes must be evaluated on a formulation-specific basis rather than generalized across entire traditional medicine systems.

1. Chinese Herbal Formulations

Several distinct Chinese herbal formulas originally developed according to traditional syndrome differentiation have been investigated in randomized clinical trials. For instance, Qinggongshoutao (QGST, a traditional palace formulation containing *Cistanche deserticola*, *Rehmannia glutinosa*, *Lycium barbarum*, *Cynomorium songaricum*, *Morinda officinalis*, *Schisandra chinensis*, *Angelica sinensis*, *Dioscorea opposita*, *Achyranthes bidentata*, and *Polygala tenuifolia*), traditionally indicated for replenishing kidney essence, was assessed in a 52-week randomized controlled trial involving 350 patients with aMCI. In this specific trial, QGST slowed progression to possible or probable AD compared with placebo and showed improvements on ADAS-cog and MMSE scores [105]. Another multi-herb preparation, Shenwu capsule (containing *Polygonum multiflorum*, *Panax ginseng*, and *Acorus tatarinowii*), was evaluated in a 24-week phase III trial in 324 patients with aMCI, showing improvements in ADAS-cog and delayed story recall scores comparable to donepezil [106]. Similarly, Bushen capsule, primarily composed of *Cistanches Herba*, *Rhizoma Alismatis*, and *P. multiflorum*, demonstrated cognitive stabilization over two years, with functional neuroimaging suggesting modulation of altered neural activity in aMCI [107]. In patients with mild AD, Yishen Huazhuo decoction (YHD)—comprising seven botanical ingredients—demonstrated greater improvement on the ADAS-cog compared with donepezil over 24 weeks [108]. In addition, a randomized, double-blind protocol for Di-Tan decoction, classically conceptualized as an intervention to “resolve phlegm,” has been proposed as an add-on therapy for AD, though efficacy outcomes remain pending [111].

2. Formulations from Other Traditional Medicine Systems

Specific multi-herb formulations from other traditional systems have also undergone preliminary clinical testing. In Traditional Persian Medicine, Davaie Loban (a fixed-combination formulation consisting of *Cyperus rotundus*, *Zingiber officinale*, *Acorus calamus*, *Piper nigrum*, and *Boswellia carterii*) was evaluated in a 12-week placebo-controlled trial in mild-to-moderate AD, demonstrating significant score improvements in ADAS-cog and CDR-SOB [109]. Within Korean traditional medicine, Jujadokseo-hwan (JDH, a classical multi-herbal formula comprising *Acorus gramineus*, *Polygala tenuifolia*, *Poria cocos*, *Alpinia oxyphylla*, *Angelica gigas*, *Rehmannia glutinosa*, *Dioscorea opposita*, and *Schisandra chinensis*), traditionally prescribed for forgetfulness, was assessed in patients with MCI; it improved disease-specific quality of life (KQOL-AD), although an economic evaluation indicated it was not cost-effective relative to placebo based on the incremental cost-effectiveness ratio [110]. Population-specific interpretation is essential. In healthy participants, the available findings primarily indicate possible modulation of selected cognitive domains and should not be interpreted as evidence for treatment or prevention of neurodegenerative disease. Evidence in individuals with subjective memory complaints remains preliminary. In MCI, several formulation-specific trials have reported improvements in cognitive outcomes, but the evidence remains heterogeneous and insufficient for generalized recommendations. In AD, clinical evidence is more limited and currently supports further evaluation primarily as an adjunctive approach rather than established monotherapy. Findings from other neurocognitive conditions should likewise be interpreted within their specific disease context and not extrapolated directly to MCI or AD.

#### 3.2.3. Methodological Limitations of Pooled Evidence

Systematic reviews and meta-analyses have reported improvements in MMSE and MoCA scores and possible reductions in progression from MCI to dementia; however, these pooled estimates should be interpreted cautiously. The certainty of the evidence is reduced by several factors. First, risk of bias remains substantial because many included trials have incomplete allocation concealment, inadequate blinding, small sample sizes, and incomplete safety reporting. Second, considerable clinical and methodological heterogeneity exists across botanical formulations, extraction and standardization procedures, treatment durations, participant characteristics, comparators, and cognitive outcome measures, contributing to inconsistency across studies. Third, imprecision is a concern because many individual trials are small and some pooled estimates are based on limited numbers of participants or events. Indirectness also limits interpretation because several studies involve heterogeneous populations, non-identical herbal formulations, or surrogate cognitive outcomes rather than clinically meaningful functional or disease-progression endpoints. Finally, publication bias and selective outcome reporting may contribute to an overrepresentation of positive findings in the available literature. Collectively, these limitations explain why statistically significant pooled effects should not be interpreted as definitive evidence of clinical efficacy or generalized across different herbal formulations. Accordingly, therapeutic signals observed in meta-analyses cannot be broadly extrapolated to entire traditional systems or untested formulas, and clinical recommendations require rigorous, formulation-specific validation. To facilitate cross-study comparison, Table 3 systematically summarizes sample size, intervention dose, treatment duration, quantitative effect estimates where available and reported adverse events or tolerability findings.

#### 3.2.4. Safety and Toxicity

1. Safety Profile, Herb–Drug Interactions, and Regulatory Considerations

Although clinical trials and systematic reviews generally suggest that botanical preparations and herbal formulations for cognitive impairment are reasonably well tolerated in the short term, comprehensive conclusions regarding long-term safety remain limited. This concern is particularly relevant in older adults, in whom multimorbidity, polypharmacy, and concurrent use of herbal and conventional medicines may increase the potential for adverse effects and herb–drug interactions [124]. Systematic reviews of Chinese herbal medicines for mild cognitive impairment indicate that adverse-event reporting remains incomplete in some trials and that gastrointestinal symptoms are among the most frequently reported adverse effects [107,108]. Individual studies have reported generally mild events, including decreased appetite, nausea, diarrhea, and constipation, in trials of formulations such as Bushen and Qinggongshoutao (QGST) [116,121]. Although serious adverse events appear uncommon and are not necessarily attributable to the herbal interventions, clinically relevant events, including hepatic abnormalities and electrolyte disturbances, have been reported in the broader herbal-medicine literature [125,126]. Accordingly, short-term tolerability should not be interpreted as evidence of long-term safety, underscoring the need for systematic assessment of organ toxicity, herb–drug interactions, and product quality.

2. Organ-Specific Toxicities and Clinical Hazards

Hepatic and renal toxicity are important considerations in long-term phytotherapy, particularly in older adults. *Polygonum multiflorum*, an ingredient of some herbal formulations used for cognitive impairment, including Bushen capsules, has been associated with herb-induced liver injury, which appears to be predominantly idiosyncratic and may involve immune-mediated mechanisms [127,128]. Several constituents, including anthraquinones and stilbene glycosides, have been investigated as potential contributors to its hepatotoxicity, although their relative clinical importance remains incompletely established [127]. Herbal medicines may also cause nephrotoxicity through multiple mechanisms, and age-related reductions in renal function may further increase vulnerability in older adults [129]. These concerns underscore the importance of appropriate hepatic and renal safety monitoring during prolonged herbal therapy.

Cardiovascular Effects and Bleeding Risks: Some herbal medicines may influence cardiovascular function, platelet activity, or coagulation and may therefore interact with conventional therapies. *Ginkgo biloba*, for example, contains ginkgolides with platelet-activating factor–antagonist activity; however, evidence that standardized Ginkgo extracts independently increase clinically significant bleeding remains inconclusive [130]. Nevertheless, potential interactions between herbal products and antiplatelet or anticoagulant drugs have been reported, particularly in older adults receiving multiple medications [124,131]. Careful medication review and monitoring are therefore advisable when herbal preparations are used concurrently with agents such as aspirin, clopidogrel, or warfarin.

3. Pharmacokinetic Herb–Drug Interactions

Potential hepatotoxicity and herb–drug interactions also warrant greater clinical attention, particularly in older adults receiving multiple medications. Although most herbal interventions evaluated in cognitive impairment trials have not shown frequent severe toxicity, herb-induced liver injury has been reported with several medicinal herbs and complex traditional formulations. Particular caution is warranted for preparations containing herbs reported hepatotoxic potential, such as *Polygonum multiflorum*, which is included in some traditional formulations discussed in this review. Published cases of herbal liver injury are predominantly hepatocellular and may occasionally be severe; however, causality assessment is complicated by variable product composition, adulteration, contamination, and incomplete characterization of multi-herbal preparations [127,132,133]. Concomitant use with conventional anti-dementia medications should also be considered carefully. Donepezil is metabolized primarily through CYP2D6- and CYP3A-related pathways, creating a theoretical potential for pharmacokinetic interactions with herbal products that modulate these enzymes [134,135]. Such interactions may alter donepezil exposure and could potentially affect therapeutic response or the risk of cholinergic adverse effects; however, the clinical relevance of these interactions remains uncertain. Available human data remain limited. For example, a small clinical study involving 14 patients with Alzheimer’s disease found that supplementation with standardized *Ginkgo biloba* extract (90 mg/day) did not significantly alter the steady-state plasma concentration of donepezil [136]. The interaction potential differs among other anti-dementia drugs: galantamine is also metabolized primarily through CYP2D6 and CYP3A4, whereas rivastigmine undergoes predominantly esterase-mediated metabolism with minimal involvement of the CYP450 system, and memantine is eliminated mainly unchanged via the kidneys [136,137,138]. These pharmacokinetic differences suggest that the likelihood and mechanisms of herb–drug interactions may vary among individual anti-dementia agents. Nevertheless, direct clinical evidence regarding interactions between herbal medicines and rivastigmine, galantamine, or memantine should be clinically monitored for possible changes in therapeutic response or adverse effects when herbal medicines are introduced, especially when complex or poorly standardized formulations are used [139]. Accordingly, patients receiving cholinesterase inhibitors or memantine should be monitored for changes in therapeutic response and adverse effects when herbal medicines are added, particularly when complex or poorly standardized formulations are used.

4. Product Quality, Identification, and Long-Term Exposure

Intrinsic and extrinsic quality issues present additional clinical hazards. For example, thujone, a constituent of Salvia officinalis and other thujone-containing botanicals, exhibits dose-dependent neurotoxicity in experimental studies, highlighting the importance of controlling potentially toxic phytochemicals and ensuring product standardization [140]. Considerable variation in thujone content has also been reported among commercial sage preparations [141,142]. Extrinsic quality concerns include botanical misidentification and substitution, contamination with toxic elements such as lead, cadmium, arsenic, and mercury, pesticide residues, and adulteration with undeclared pharmaceutical substances [143,144,145,146,147]. These concerns are particularly relevant to chronic therapy, because clinical trials of herbal interventions for cognitive impairment and dementia are generally of limited duration, often extending from several months to approximately one year [148]. Consequently, available trials provide limited information on cumulative or delayed adverse effects associated with long-term use, underscoring the need for longer-term safety surveillance and adequately powered longitudinal studies.

Clinical trials and systematic reviews suggest that herbal medicines and phytochemical-rich preparations for cognitive impairment are generally well tolerated; however, safety conclusions remain limited by inconsistent adverse event reporting, short intervention periods, and heterogeneity of herbal products. In placebo-controlled trials of CHM for MCI, a recent meta-analysis of 13 randomized trials involving 1043 participants found no significant difference in adverse events between CHM and placebo, with gastrointestinal symptoms being the most commonly reported events [108]. Similarly, a broader systematic review of 49 randomized trials reported no statistically significant difference in adverse events between CHM and control groups, although the overall methodological quality of included trials was relatively low [107]. In trials of *Bacopa monnieri*, safety reporting was not the main focus, but the reviewed studies were generally conducted using standardized extracts for 12 weeks, with dropout rates varying across studies; thus, more systematic adverse event monitoring is still needed [149]. Individual clinical trials of traditional herbal formulations also provide supportive but formulation-specific safety information. In the phase III trial of Shenwu capsule for amnestic MCI, adverse events were less frequent in the herbal therapy group than in the donepezil group, and the treatment was reported to be safe and well tolerated [122]. Bushen capsule was evaluated over a two-year period in patients with amnestic MCI, with mild adverse events such as decreased appetite, constipation, and nausea reported in both herbal and placebo groups [150]. Jujadokseo-hwan improved quality-of-life outcomes in patients with MCI, although the study focused primarily on economic evaluation rather than detailed toxicity assessment [120]. For QGST, the 52-week randomized trial in amnestic MCI provides relatively long-term clinical evidence, but further detailed safety evaluation remains necessary for broader application [121]. For AD, safety evidence is more cautious. A systematic review of CHM for AD reported that adverse events were documented in some trials but not consistently analyzed across all studies; gastrointestinal symptoms were common, and serious events such as hepatic function impairment, cardiovascular events, acute heart failure, cholelithiasis, and hypokalemia were reported in some cases [109]. In clinical trials of Yishen Huazhuo decoction and Davaie Loban, cognitive benefits were reported, but larger studies with more rigorous hepatic, renal, cardiovascular, and herb–drug interaction monitoring are still needed [117,123]. Safety concerns are also relevant for specific botanical groups, such as *Salvia* spp., where thujone-containing preparations require quality control, and quercetin-enriched culinary herb formulations, where short-term tolerability was acceptable but long-term safety remains unclear [101,104].

### 3.3. Mechanisms of Action of Herbal Medicines in Cognitive Impairment

Selected herbal extracts and their bioactive constituents may mitigate cognitive impairment through distinct or complementary mechanisms that address several interconnected pathological processes, rather than functioning as a single pharmacological entity. One of the most consistently reported properties among certain botanical preparations is the modulation of cholinergic neurotransmission through the inhibition of acetylcholinesterase and, in some cases, butyrylcholinesterase. By reducing the enzymatic degradation of acetylcholine, herbal extracts and their constituents may enhance synaptic cholinergic signaling involved in attention, learning, and memory. Some preparations additionally increase choline acetyltransferase activity or modulate muscarinic receptor expression, further supporting cholinergic function. This mechanism has been observed in both enzyme-based studies and animal models of scopolamine-induced cognitive impairment.

Antioxidant properties and mitochondrial modulation represent another major area of investigation documented for particular botanical agents. Importantly, general biochemical antioxidant activities must be distinguished from direct, functional mitochondrial preservation. At the biochemical level, certain plant extracts and polyphenolic compounds reduce reactive oxygen species (ROS), inhibit lipid peroxidation, and upregulate endogenous enzymatic defenses (such as glutathione, superoxide dismutase, catalase, and Nrf2/HO-1 signaling). Beyond these radical-scavenging actions, a smaller subset of preparations has been specifically evaluated for functional mitochondrial endpoints, showing preservation of mitochondrial membrane potential ($\Delta \Psi_{m}$), maintenance of ATP production, protection of respiratory-chain complex activity, and regulation of apoptosis-related proteins such as Bax and caspase-3.

Because oxidative stress and mitochondrial dysfunction contribute to synaptic failure and neuronal loss, preservation of mitochondrial integrity may be central to the neuroprotective effects of selected botanical preparations. Specific herbal preparations have also been demonstrated to suppress neuroinflammation by reducing microglial activation and the production of pro-inflammatory mediators, including TNF-α, IL-1β, IL-6, nitric oxide, and NF-κB-related signaling. Inhibition of MAPK pathways, including JNK, ERK, and p38, has also been reported for individual extracts. In parallel, discrete phytochemicals modulate pathways implicated in AD pathogenesis in experimental models by reducing amyloid-β production or aggregation, promoting fibril disassembly and autophagic clearance, shifting amyloid precursor protein processing toward the non-amyloidogenic pathway, and suppressing tau hyperphosphorylation through regulation of GSK-3β, protein phosphatase 2A, and related signaling pathways. Although experimental studies indicate that selected herbal extracts and phytochemicals can modulate amyloidogenic processing, Aβ aggregation or clearance, and tau phosphorylation, these findings are derived predominantly from cellular and animal models. Such mechanistic effects should not be interpreted as evidence of disease-modifying efficacy in humans. Current clinical studies have largely focused on cognitive outcomes, and longitudinal biomarker-based evidence demonstrating that herbal interventions reduce amyloid or tau pathology, alter validated disease biomarkers, or slow the underlying progression of MCI or AD remains insufficient. Accordingly, herbal medicines cannot presently be classified as disease-modifying therapies. In addition, individual herbal extracts promote neuronal survival, differentiation, neurite outgrowth, synaptogenesis, and synaptic plasticity through neurotrophic pathways such as BDNF/TrkB and CREB signaling. Enhancement of these pathways may support long-term potentiation, hippocampal neurogenesis, and restoration of neuronal connectivity. Taken together, the available evidence indicates that individual herbal products do not universally engage all pathways; rather, distinct extracts and phytochemicals differentially modulate subsets of these targets, which may collectively encompass cholinergic signaling, oxidative stress, mitochondrial function, neuroinflammation, amyloid and tau pathology, apoptosis, and neurotrophic signaling.

Importantly, the interpretation of these proposed mechanisms must also consider pharmacokinetic plausibility. Demonstration of enzyme inhibition, signaling modulation, cytoprotection, or other biological activity in biochemical or cellular models does not establish that the responsible constituent reaches comparable concentrations in vivo. Mechanistic interpretation should therefore distinguish demonstrated biological activity from achievable systemic exposure, CNS exposure following blood–brain barrier penetration, the activity of circulating or brain-available metabolites, and direct evidence of target engagement. In some cases, metabolites rather than the parent phytochemicals may represent the biologically relevant species. Accordingly, mechanisms observed only at concentrations substantially exceeding achievable systemic or CNS exposure should be considered hypothesis-generating unless supported by pharmacokinetic, biodistribution, and target-engagement data. Where such information is unavailable, the proposed mechanism should be regarded as biologically plausible but pharmacologically unconfirmed. These diverse, mechanistic pathways identified across evaluated botanical candidates are summarized in Figure 2. However, their biological and translational relevance depends not only on the botanical species, extract composition, dose, and disease model, but also on the bioavailability of active constituents or metabolites, achievable systemic and CNS exposure, and evidence of target engagement.

### 3.4. Challenges and Knowledge Gaps

Despite increasing evidence supporting the neuroprotective potential of herbal medicines, substantial methodological limitations continue to restrict the interpretation and translation of current findings. Much of the preclinical evidence is derived from enzyme-based assays, neuronal cell lines, and acute pharmacologically induced animal models, particularly scopolamine-induced amnesia. Although these models are useful for investigating cholinergic dysfunction and short-term memory impairment, they do not adequately reproduce the progressive amyloid, tau, vascular, metabolic, and neurodegenerative alterations associated with MCI and AD.

Model selection also requires greater mechanistic justification. The widely used scopolamine-induced amnesia model is useful for rapid screening of interventions targeting cholinergic dysfunction and short-term learning and memory deficits, but its effects are largely transient, and it does not reproduce the progressive neurodegeneration or the full spectrum of amyloid, tau, vascular, and metabolic abnormalities observed in human AD. Conversely, transgenic models can reproduce selected amyloid- or tau-related features but often represent familial genetic forms of AD and may not adequately model the multifactorial nature of late-onset sporadic disease [151,152]. Future studies should therefore select models according to the biological question being addressed rather than relying on a single convenient paradigm. Scopolamine models remain appropriate for investigating cholinergic mechanisms and initial cognitive screening, whereas amyloid- or tau-focused transgenic models are preferable for evaluating disease-specific proteinopathy. Intracerebroventricular streptozotocin models may provide complementary information on brain insulin resistance and metabolic features associated with sporadic AD, while vascular or chronic hypoperfusion models are more appropriate when cerebrovascular mechanisms are the primary target. For interventions proposed to have broad disease-modifying effects, confirmation across more than one mechanistically distinct model, preferably incorporating aged animals and relevant metabolic or vascular comorbidities, would substantially strengthen translational confidence [151,152,153]. Across these models, standardized behavioral, biochemical, molecular, and neuropathological endpoints should also be employed to facilitate reproducibility and cross-study comparison. These model-related limitations are compounded by pharmacokinetic barriers that may further contribute to the discrepancy between robust preclinical effects and inconsistent clinical outcomes. Many phytochemicals exhibiting neuroprotective activity experimentally have limited oral bioavailability, extensive metabolism, or insufficient blood–brain barrier penetration, resulting in systemic or brain exposures substantially lower than those used in vitro or in animal studies. Species-specific differences in metabolism, pharmacokinetics, and systemic exposure further complicate extrapolation of effective doses and mechanisms from animal models to humans. Together, these pharmacokinetic and disease-model differences help explain why promising preclinical findings do not necessarily translate into clinically meaningful benefits.

Another major challenge is the considerable heterogeneity of herbal interventions. Differences in botanical identity, plant part, geographical origin, extraction method, phytochemical composition, dose, and treatment duration make direct comparison between studies difficult. Although botanical and chemical standardization characteristics have now been systematically summarized in the evidence tables where reported, substantial gaps remain. Many primary studies do not provide sufficient information on botanical authentication, voucher specimens, geographical origin, extraction ratios or yields, chemical fingerprints, quantitative marker compounds, or batch-to-batch consistency. This incomplete reporting limits reproducibility and complicates direct comparison across studies. These quality-control issues are particularly relevant to frequently investigated herbs such as *Bacopa monnieri*, *Crocus sativus*, and *Panax ginseng*. For *Bacopa monnieri*, reproducibility requires verified botanical identity, specification of the plant part and cultivation source, and chemical standardization of the extract, particularly its bacoside-related constituents. Considerable variation in *Bacopa monnieri* preparations has been attributed to differences in plant source, cultivation conditions, extraction solvent, and the type and content of standardized bacosides, which can complicate comparison across experimental and clinical studies [154]. For saffron (*Crocus sativus*), authentication of the dried stigmas and quantitative assessment of characteristic constituents, particularly crocins, picrocrocin, and safranal, are important quality-control parameters. Saffron composition may vary according to geographical origin, post-harvest drying, and storage conditions, while its high commercial value also creates a substantial risk of adulteration; therefore, chromatographic or spectroscopic fingerprinting should complement conventional quality specifications when extracts are intended for research or clinical use [155,156]. For *Panax ginseng*, species identity, geographical origin, cultivation conditions, plant age and part, and processing method should be documented, together with a quantitative ginsenoside profile. Processing is especially important because fresh, white, red, fermented, and other processed ginseng products differ substantially in their ginsenoside composition [157,158]. Accordingly, apparently contradictory pharmacological or clinical findings between preparations may reflect genuine differences in phytochemical composition, dose, extraction efficiency, processing-induced transformation, stability, and bioavailability rather than simply experimental inconsistency. In addition, pharmacokinetic information, oral bioavailability, metabolite profiles, blood–brain barrier penetration, and herb–drug interactions remain poorly characterized. Greater integration of pharmacognostic authentication, chemical standardization, metabolomics, pharmacokinetic–pharmacodynamic analysis, and batch-to-batch quality control is therefore essential, particularly for complex multi-herbal formulations. Clinical evidence remains promising but inconclusive because many trials involve small sample sizes, short intervention periods, heterogeneous populations, variable diagnostic criteria, and inconsistent cognitive outcome measures. The clinical literature may also be affected by publication bias and selective outcome reporting, particularly because many available trials are small and may preferentially report statistically significant cognitive or biomarker outcomes. This limitation further reduces confidence in the apparent consistency of positive findings. Several studies have also focused on healthy adults or individuals with subjective memory complaints rather than patients with biomarker-confirmed MCI or AD. Moreover, long-term safety, clinically meaningful functional outcomes, disease progression, and rare adverse events are insufficiently evaluated. Future multicenter randomized controlled trials should use standardized herbal products, rigorous blinding and allocation procedures, longer follow-up, biomarker-supported diagnosis, harmonized cognitive and functional endpoints, and systematic monitoring of hepatic, renal, cardiovascular, and herb–drug interaction risks. Pharmacokinetic–pharmacodynamic characterization and herb–drug interaction studies also remain important priorities, particularly for complex multi-herbal formulations. Consequently, molecular effects observed at high experimental concentrations cannot be assumed to occur in the human brain without supporting exposure and target-engagement data.

### 3.5. Future Perspectives

Future research on herbal medicines for cognitive impairment must transition from exploratory bioactivity screening toward a rigorous translational framework that prioritizes chemical reproducibility, pharmacokinetic plausibility, clinical trial rigor, and comprehensive safety monitoring. First and foremost, establishing product consistency remains the primary prerequisite for translational validity. Future studies must enforce strict botanical authentication, documented herbarium voucher specimens, and standardized extraction protocols to eliminate batch-to-batch variation. Chemical standardization should extend beyond single-marker profiling to include comprehensive multi-marker quantification and validated chromatographic or spectroscopic fingerprinting. Concurrently, precise dose–response characterization and systematic exposure assessments are required to define reproducible therapeutic windows, ensuring that preclinical experimental doses are physiologically relevant and scalable to humans.

Second, robust pharmacokinetic and biodistribution studies must be prioritized to establish biological plausibility. Many promising phytochemicals suffer from poor oral bioavailability, extensive first-pass metabolism, or rapid systemic clearance. Investigating the pharmacokinetic profiles of parent compounds, characterizing active circulating metabolites, and quantitatively demonstrating blood–brain barrier (BBB) penetration is critical to verify that active constituents achieve neuroprotective concentrations at target sites within the central nervous system and engage the proposed molecular targets in vivo. Advanced analytical tools, including metabolomics and network pharmacology, can serve as secondary exploratory methods to identify active metabolite fractions and map multi-target interactions, provided they are coupled with empirical pharmacokinetic validation.

Third, the clinical translation of herbal interventions requires adequately powered, multi-center randomized controlled trials (RCTs) that test chemically well-characterized, batch-controlled formulations against placebo or active comparators. Future trials should adopt biomarker-supported diagnostic criteria (e.g., amyloid, tau, or neurofilament light chain markers) and follow-up durations of sufficient length to detect alterations in disease trajectory. Crucially, study designs must move beyond isolated neuropsychological test scores to measure clinically meaningful endpoints, including validated activities of daily living (ADL), caregiver burden, and health-related quality of life.

Finally, in an aging population characterized by multimorbidity and extensive polypharmacy, systematic safety evaluations must become a central pillar of clinical development. Dedicated, prospective assessments of long-term safety and organ toxicities—specifically hepatic and renal surveillance—are essential. Given that patients frequently receive concurrent antidementia agents, antihypertensives, and antithrombotic therapies, systematic in vitro and in vivo assessments of cytochrome P450/P-glycoprotein modulation and formal herb–drug interaction studies are imperative to prevent adverse pharmacological outcomes. Ultimately, the most viable translational path for herbal medicines lies in rigorously validated, batch-standardized adjunctive or preventative strategies within multimodal clinical management, rather than unstandardized monotherapies.

## 4. Conclusions

Preclinical studies provide valuable mechanistic evidence that selected herbal extracts, standardized preparations, traditional formulations, and their bioactive constituents can modulate biological pathways relevant to cognitive impairment, including cholinergic neurotransmission, oxidative stress, neuroinflammation, functional mitochondrial endpoints, amyloid- and tau-related pathologies, and neurotrophic signaling. Accordingly, mechanistic and preclinical findings should be viewed as hypothesis-generating and should not be interpreted as evidence of clinical efficacy unless supported by appropriately designed human trials. Furthermore, clinical trials evaluating specific standardized extracts and defined multi-herb formulations have reported preliminary improvements in cognitive metrics, such as memory, attention, MMSE, MoCA, and ADAS-cog scores. However, the strength and generalizability of these findings remain heavily constrained by substantial heterogeneity in botanical composition, extraction techniques, phytochemical yields, experimental models, study populations, dosing regimens, and outcome measures. Because the available evidence varies considerably among individual plants, preparations, and clinical cohorts, herbal medicines cannot be evaluated or characterized as a unified intervention class. Consequently, no generalized therapeutic recommendation can currently be made for herbal medicines as a class for the prevention or treatment of cognitive impairment. At present, positive clinical signals remain strictly formulation- and extract-specific, with insufficient evidence to support botanical products as established monotherapies or replacements for conventional clinical management. The most plausible therapeutic role for individually validated preparations lies in carefully evaluated adjunctive or preventive strategies within multimodal care, particularly in individuals with mild cognitive impairment or early-stage cognitive decline. Future clinical translation of any specific botanical candidate will depend strictly on rigorous botanical authentication, comprehensive phytochemical standardization, robust pharmacokinetic and central nervous system exposure verification, adequately powered multicenter trials assessing clinically meaningful functional endpoints (such as activities of daily living), and extensive evaluations of long-term safety and herb–drug interactions in older adults with polypharmacy.

## Figures and Tables

**Figure 1:**
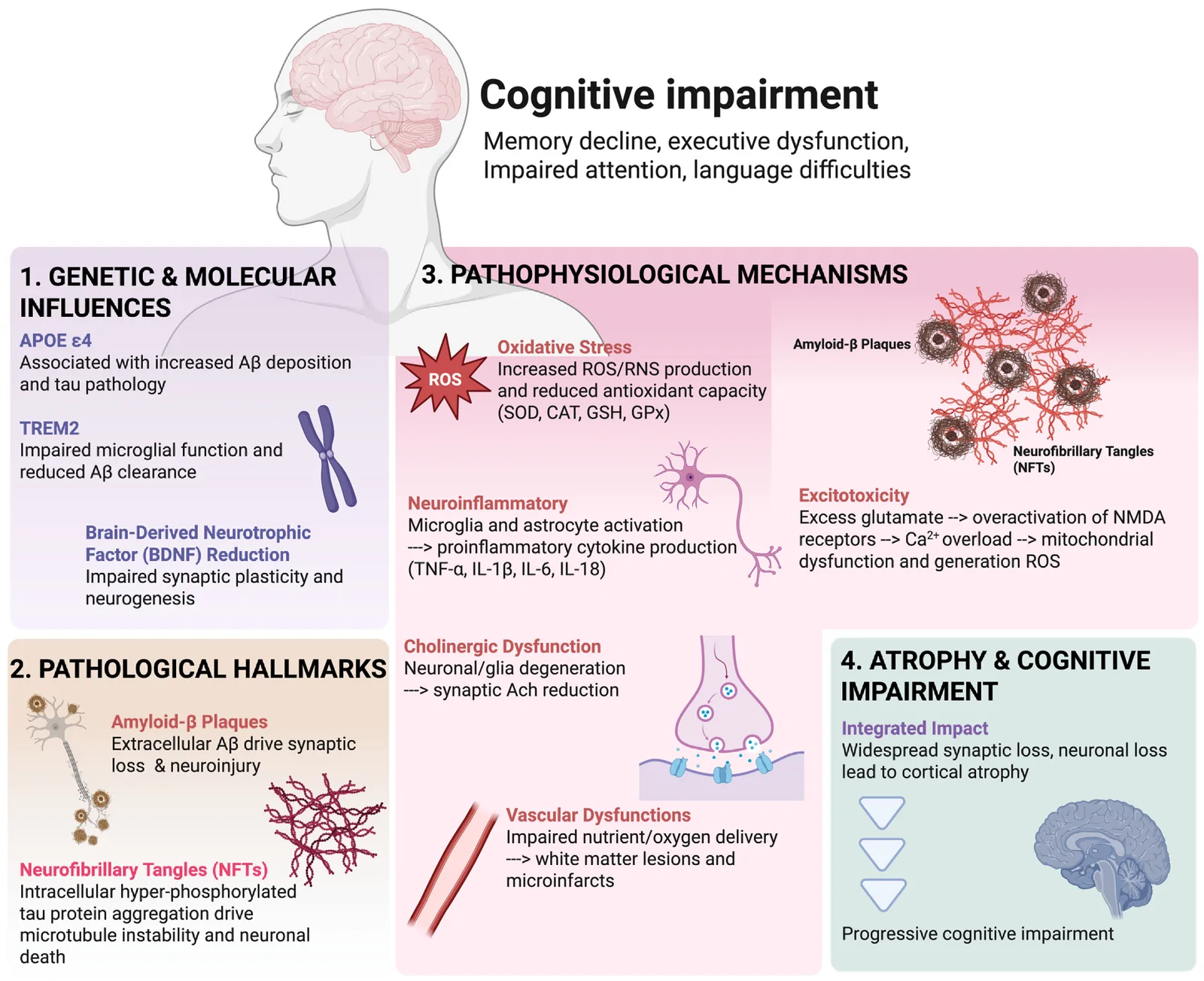
Major molecular, pathological, and pathophysiological mechanisms underlying cognitive impairment. Created using BioRender by Aekkhaluck Intharuksa. Available at https://BioRender.com/tj4qwpu (accessed on 19 August 2026).

**Figure 2:**
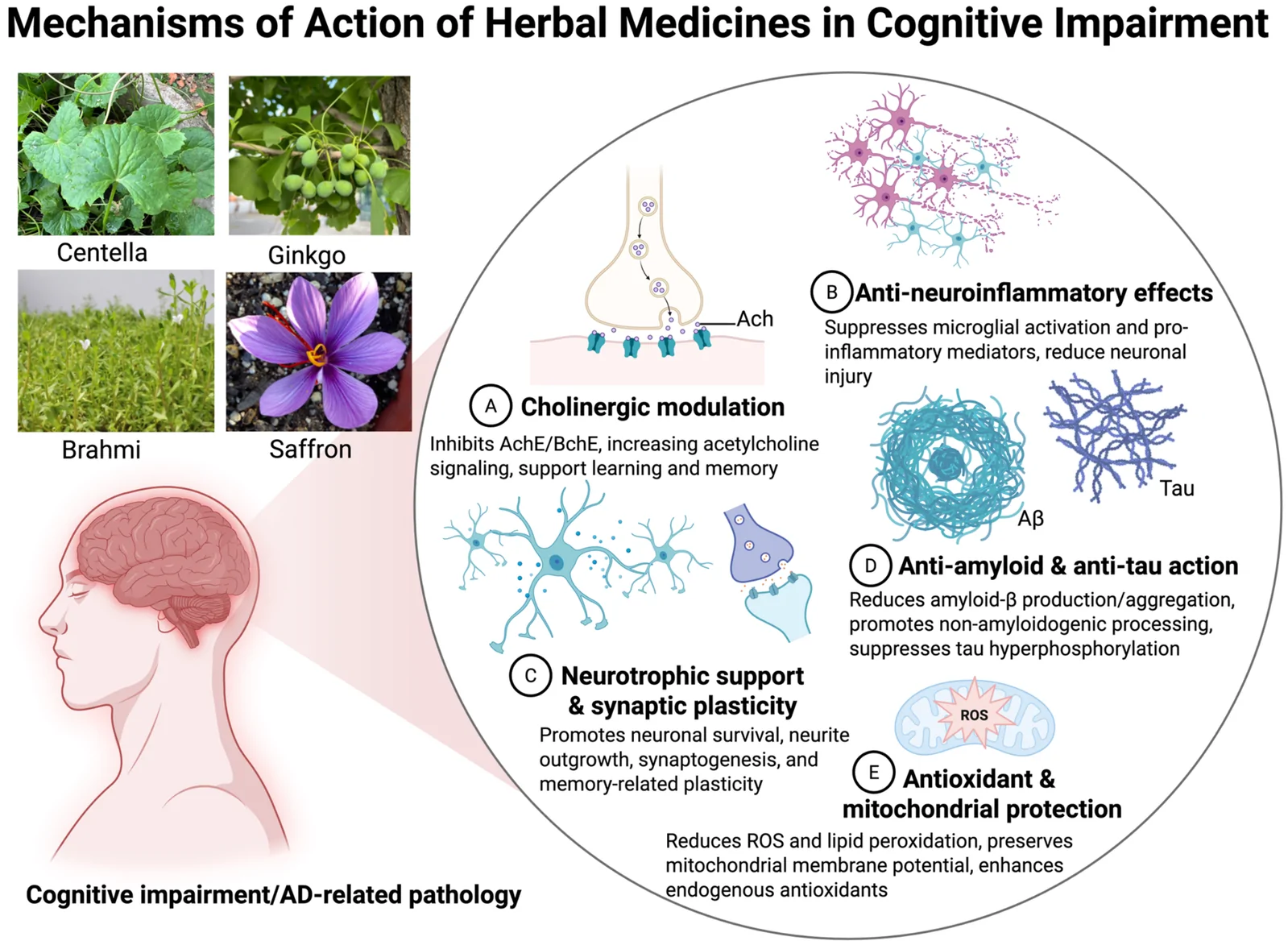
Major neuroprotective mechanisms of herbal medicines against cognitive impairment. Representative medicinal plants, including *Centella asiatica* (Centella), *Ginkgo biloba* (Gingko), *Bacopa monnieri* (Brahmi), and *Crocus sativus* (saffron), may exert cognitive-protective effects through multiple mechanisms, including (A) cholinergic modulation, (B) anti-neuroinflammatory activity, (C) enhancement of neurotrophic support and synaptic plasticity, (D) anti-amyloid and anti-tau effects, and (E) antioxidant and mitochondrial protection. These complementary actions may collectively reduce neuronal damage and cognitive decline associated with AD-related pathology. Created using BioRender by Aekkhaluck Intharuksa. Available at https://BioRender.com/1nh2z58 (accessed on 19 August 2026).

**Table 2:** In vivo evidence of herbal medicines and traditional herbal formulations for cognitive impairment.

Herbal Intervention	Botanical Authentication and Chemical Standardization	In Vivo Model and Dose	Main Outcomes	Proposed Mechanisms
*Zanthoxylum alatum* [83]	Seed hydroethanolic extract; marker standardization NR	Scopolamine-induced amnesia in rats; 50, 100, and 200 mg/kg; radial arm maze	Reduced working memory and reference memory errors	Downregulated AChE, NFκB, Tau, Bax, and caspase-3; upregulated Nrf2, HO-1, PP2A, BDNF, and TrkB; reduced TNF-α and IL-1β and restored IL-10
*Hibiscus abelmoschus* [86]	Seed ethanol extract; marker standardization NR	Scopolamine-induced amnesia in mice; 200 and 400 mg/kg	Improved Morris water maze, elevated plus maze, and passive avoidance performance	Reduced lipid peroxidation; increased GSH and CAT; lowered cholesterol and triglycerides
*Silybum marianum* [95]	Seed methanol extract; HPLC identified quercetin, rutin, and morin	Scopolamine-induced memory impairment in mice; 50, 100, and 200 mg/kg	Improved Y-maze spontaneous alternation and NORT discrimination index	AChE/BChE inhibition; antioxidant activity; HPLC-detected quercetin, rutin, and morin; docking-supported AChE binding
*Willughbeia cochinchinensis* [87]	Wood preparation; quantitative marker standardization NR	Scopolamine-induced cognitive deficits in mice; 100, 150, and 200 mg/kg	Prevented deficits in Y-maze, Morris water maze, and novel object recognition	Anti-AChE and anti-BChE activities
*Huperzia serrata* [90]	Whole-plant preparation; caffeic acid, ferulic acid, and huperzine A identified	Scopolamine-induced cognitive dysfunction in mice; 100 mg/kg, p.o.	Improved arm alternation and latency	AChE inhibition; reduced lipid peroxidation; caffeic acid, ferulic acid, and huperzine A identified
*Huperzia serrata* alkaloid fraction [53]	Total alkaloid fraction from aerial parts; formal marker standardization NR	Scopolamine-induced memory impairment in mice; compared with huperzine A	Improved Y-maze and Morris water maze performance	Reduced AChE and MDA; increased SOD and CAT in hippocampus and cortex
*Dioscorea opposita* [96]	Rhizome CHCl_3_-soluble fraction; marker standardization NR	Scopolamine-induced amnesia in mice; acute 200 mg/kg and repeated 50 mg/kg	Improved spatial learning and memory in Morris water maze and passive avoidance test	Neuroprotection against glutamate- and H_2_O_2_-induced toxicity in cortical neurons
*Orthosiphon stamineus* [85]	Authenticated; voucher #11009; standardized extract containing rosmarinic acid, sinensetin, eupatorin, and TMF	ICV-STZ-induced AD-like rat model	Reversed learning and memory dysfunction	Downregulated APP, MAPT, NFκB, GSK3α, and GSK3β; standardized to rosmarinic acid, sinensetin, eupatorin, and TMF
*Evolvulus alsinoides* and *Centella asiatica* [97]	Whole-plant ethanol extracts; marker standardization NR	Scopolamine-induced amnesia in Swiss albino mice; 250 and 500 mg/kg, alone and combined	Improved spatial memory formation in elevated plus maze and Morris water maze	Reduced AChE; improved antioxidant status; reduced IL-1β, IL-6, and TNF-α
*Nigella sativa*, rosemary, sage, and ginseng [84]	Defined plant-part extracts; detailed marker standardization NR	Long-term scopolamine-induced AD-like model in rats; NS 400 mg/kg, rosemary 200 mg/kg, sage 600 mg/kg, ginseng 200 mg/kg	All four extracts moderated cognitive–behavioral impairment in NOR, Y-maze, and Morris water maze	Reduced oxidative stress; improved neurotransmitter status; reduced AChE gene expression; attenuated neurodegenerative histopathology
Ga-Mi Gongjindan [91]	Defined multi-herb formulation; quantitative marker standardization NR	Scopolamine-induced cognitive impairment in mice; 100, 200, and 400 mg/kg	Improved spatial learning and memory in Morris water maze	Reduced AChE; increased ChAT; suppressed oxidative stress and inflammation; activated BDNF/CREB signaling; increased TrkA, TrkB, and M1 mAChR expression

NR, not reported in the original study. For isolated compounds, extract-level standardization was considered not applicable.

## Data Availability

No new data were created or analyzed in this study. Data sharing is not applicable to this article.

## Abbreviations

The following abbreviations are used in this manuscript:

AD	Alzheimer’s disease
ADAS-cog	Alzheimer’s Disease Assessment Scale–Cognitive Subscale
ACh	Acetylcholine
AChE	Acetylcholinesterase
APP	Amyloid precursor protein
Aβ	Amyloid-β
BDNF	Brain-derived neurotrophic factor
BChE	Butyrylcholinesterase
BBB	Blood–brain barrier
CAT	Catalase
CDK5	Cyclin-dependent kinase 5
CHM	Chinese herbal medicine
ChEI	Cholinesterase inhibitor
CREB	cAMP response element-binding protein
ERK	Extracellular signal-regulated kinase
GSH	Glutathione
GSK-3β	Glycogen synthase kinase-3β
IL	Interleukin
LPS	Lipopolysaccharide
LTP	Long-term potentiation
MAPK	Mitogen-activated protein kinase
MCI	Mild cognitive impairment
MMSE	Mini-Mental State Examination
MoCA	Montreal Cognitive Assessment
NF-κB	Nuclear factor kappa B
NFT	Neurofibrillary tangle
NMDA	N-methyl-D-aspartate
Nrf2	Nuclear factor erythroid 2-related factor 2
ROS	Reactive oxygen species
SOD	Superoxide dismutase
TNF-α	Tumor necrosis factor-α
TrkB	Tropomyosin receptor kinase B
TREM2	Triggering receptor expressed on myeloid cells 2
